# Mechanisms underlying the interactions and adaptability of nitrogen removal microorganisms in freshwater sediments

**DOI:** 10.1007/s44307-024-00028-6

**Published:** 2024-06-17

**Authors:** Dandan Zhang, Huang Yu, Xiaoli Yu, Yuchun Yang, Cheng Wang, Kun Wu, Mingyang Niu, Jianguo He, Zhili He, Qingyun Yan

**Affiliations:** 1 Marine Synthetic Ecology Research Center, Southern Marine Science and Engineering Guangdong Laboratory (Zhuhai), School of Environmental Science and Engineering/Life Sciences/Ecology, Guangdong Provincial Observation and Research Station for Marine Ranching in Lingdingyang Bay, China-ASEAN Belt and Road Joint Laboratory On Mariculture Technology, State Key Laboratory for Biocontrol, Sun Yat-Sen University, Zhuhai, 519082 China; 2 School of Resources Environment and Safety Engineering, Key Discipline Laboratory for National Defense for Biotechnology in Uranium Mining and Hydrometallurgy, University of South China, Hengyang, 421001 China

**Keywords:** Eutrophic ecosystems, Metagenomics, Metatranscriptomics, Microbial function, N removal microorganisms

## Abstract

Microorganisms in eutrophic water play a vital role in nitrogen (N) removal, which contributes significantly to the nutrient cycling and sustainability of eutrophic ecosystems. However, the mechanisms underlying the interactions and adaptation strategies of the N removal microorganisms in eutrophic ecosystems remain unclear. We thus analyzed field sediments collected from a eutrophic freshwater ecosystem, enriched the N removal microorganisms, examined their function and adaptability through amplicon, metagenome and metatranscriptome sequencing. We found that the N removal activities could be affected through potential competition and inhibition among microbial metabolic pathways. High-diversity microbial communities generally increased the abundance and expression of N removal functional genes. Further enrichment experiments showed that the enrichment of N removal microorganisms led to a development of simplified but more stable microbial communities, characterized by similar evolutionary patterns among N removal microorganisms, tighter interactions, and increased adaptability. Notably, the sustained provision of NH_4_^+^ and NO_2_^−^ during the enrichment could potentially strengthen the interconnections among denitrification, anaerobic ammonium oxidation (anammox) and dissimilatory nitrate reduction to ammonium (DNRA) processes. Moreover, the identification of shared metabolic traits among denitrification, anammox and DNRA implies important cooperative associations and adaptability of N removal microorganisms. Our findings highlight the microbial interactions affect the adaptive strategies of key microbial taxa involved in N removal.

**Supplementary Information:**

The online version contains supplementary material available at 10.1007/s44307-024-00028-6.

## Introduction

The microbial nitrogen (N) removal is a key ecological process controlling the N status in the global carbon cycle. It is mainly regulated by the anaerobic ammonium oxidation (anammox), denitrification, and dissimilatory nitrate (NO_3_^−^) reduction to ammonium (DNRA) (Wei et al. 2022). The transformation of NO_3_^−^ to nitrite (NO_2_^−^) is functionally identical in both DNRA and denitrification processes (You et al. 2020). However, the DNRA can further reduce NO_2_^−^ to ammonium (NH_4_^+^), which is another substrate for anammox bacteria. Previous studies suggested that high DNRA activities might cause low N removal in anammox bioreactors (Wang et al. 2022). In contrast, the denitrifying bacteria reduce NO_2_^−^ to nitrogen gas (N_2_), and thus are regarded as competitors of anammox bacteria (Dong et al. 2011). Additionally, the canonical heterotrophic denitrifying bacteria can use organic carbon in wastewater to keep NO_2_^−^ lower than the toxic concentration of anammox bacteria (Ma et al. 2020). Currently, there is no pure culture of anammox bacteria, possibly due to their strong dependence on other microorganisms (Cao et al. 2017). However, the DNRA and denitrification bacteria may be a double-edged sword when coexist with anammox bacteria. Thus, further studies are urgently needed to elucidate the interactions among the DNRA, denitrification and anammox bacteria to enhance the N removal in eutrophic ecosystems.

The average annual N loss contributed by the denitrification and DNRA has greatly exceeded that of anammox bacteria (Roland et al. 2018), which exhibit a preference for specific ecological niches within particular aquifers (Roland et al. 2018). Moreover, the diversity of anammox bacteria tends to decrease in summer, although this may be ecosystem-specific (Qian et al. 2018). The activity of anammox is also commonly constrained to distinct layers of water or sediments, as well as in specific soil depths (Humbert et al. 2010). For example, the substantial influx of organic carbon in coastal ecosystems may induce anoxic conditions and favor the denitrification in surface sediments. However, the denitrification rate always decreases with sediment depths (Wu et al. 2021). In the past decades, more and more studies realized that the microbial N removal capability could be influenced by various environmental factors (Li et al. 2021; Tan et al. 2019; Wu et al. 2019a; Zhang et al. 2020), and research efforts have especially focused on specific taxa involved in N transformation. However, environmental effects on the distribution of denitrifying, DNRA and anammox bacteria in different ecosystem remain largely inconsistent (Yang et al. 2022; Yuan et al. 2021). Environmental factors, such as substrate availability, pH, temperature, and oxygen levels, significantly influence the adaptive capacity of N removal microorganisms. Thus, it is urgently necessary to comprehend these diverse N potentials to decipher the N removal mechanisms in eutrophic ecosystems (Wang et al. 2019; Lawson et al. 2017).

The N removal is generally mediated by different microorganisms, which exhibit complex interactions and adaptive capacities in eutrophic ecosystems (Canfield et al. 2010). For example, microbial abundance and diversity were important in controlling denitrification, DNRA and anammox rates (Ma et al. 2020). Also, the N removal microorganisms often exhibit complex succession, and different groups may dominate at stages with specific resources (Zheng et al. 2016). However, the effects of microbial interactions, including competition, on the N removal functions have been largely overlooked (Kartal et al. 2007). Thus, understanding microbial interactions is also critical for clarifying the composition and function of the N removal microorganisms (Chen et al. 2016; Wang et al. 2017; Xu et al. 2018). Due to high impacts and complex interactions of bacterial community on the N metabolism (Wang et al., 2023), a stable and simple system is especially necessary to verify the N removal mechanisms in natural ecosystems (Widder et al. 2016). With the reactor system established to increase N removal, it is possible to filter out the influence of environmental factors, providing a simplified perspective for uncovering bacterial interactions and potential metabolic mechanisms. The enrichment effectively unleashes the metabolic potentials of specific pathways (Banerjee et al. 2016).

This study aims to clarify the ecological adaptation strategies of the N removal microorganisms in freshwater ecosystems. We integrated metagenome and metatranscriptome sequencing to investigate the relationships among the N function, bacterial community and abiotic factors focusing on an aquaculture system. We hypothesized that the efficiency of N removal in eutrophic ecosystems is influenced by the functional diversity and adaptability of N removal microorganisms, which are shaped by environmental factors, microbial interactions, and competitive dynamics. To test this hypothesis, we established a lab-scale reactor with freshwater sediments to enrich the N removal microorganisms under stable conditions. The relatively lower microbial diversity of the enriched microbial communities, compared to in situ communities, provides a simpler perspective for verifying the effects of biotic interactions. This study elucidates the interactions among microbes and their coupled N removal mechanisms through multi-omics approaches and functional assays. It provides novel insights that broaden our understanding of the function and adaptability of N removal microorganisms, which also shows important implications for managing high N levels in eutrophic ecosystems.

## Materials and Methods

### In situ* sampling and bioreactor operation*

**Fig. 1:**
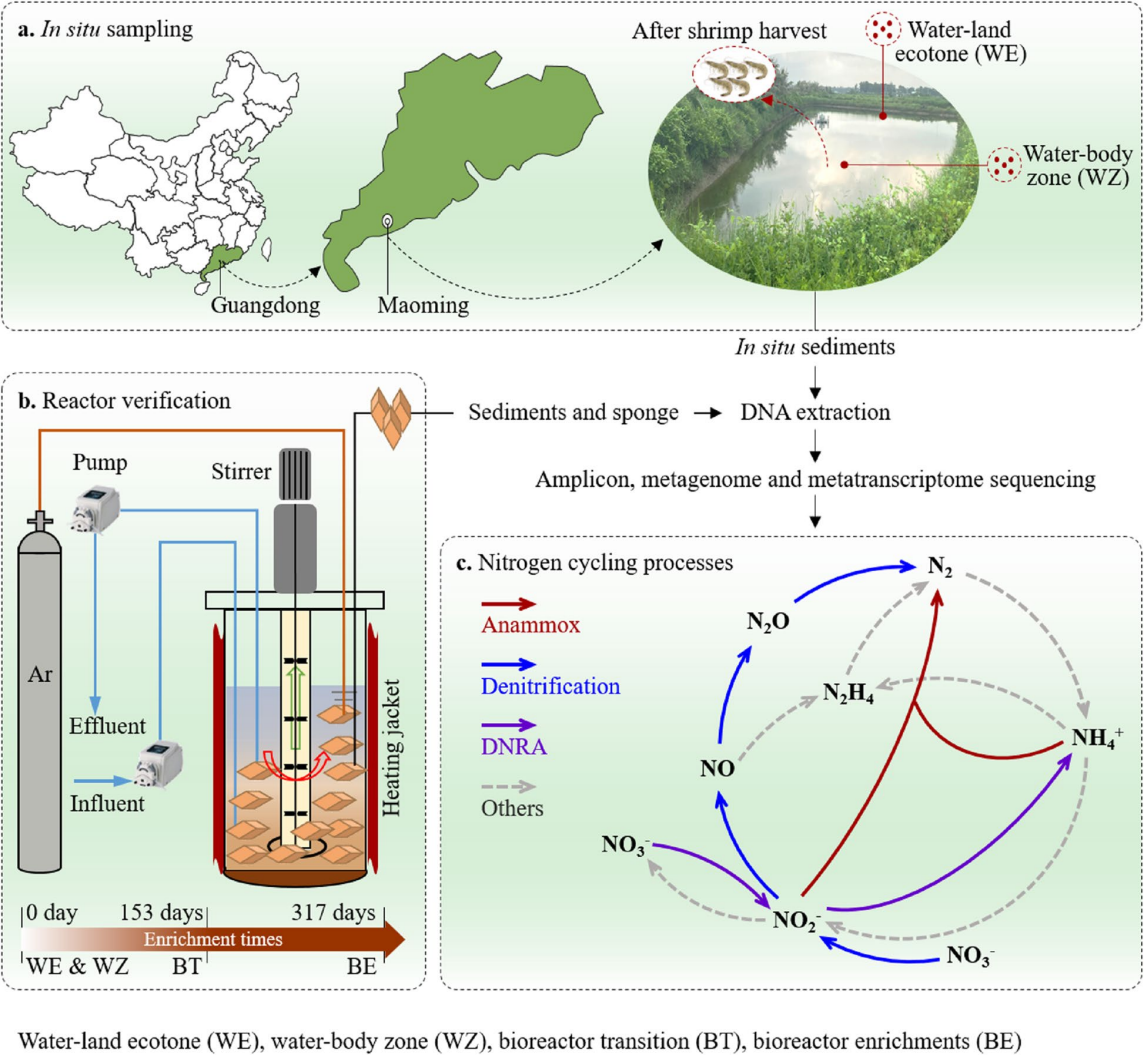
The experimental design with in situ sampling and reactor enrichment for studying the microbial nitrogen (N) removal. **a** Sampling locations and the in situ sites sampled in the aquaculture pond in Maoming. **b** The schematic design of the N removal reactor. **(c)** The N-related transformation by highlighting the N removal processes. DNRA-dissimilatory nitrate reduction to ammonium

This study focused on microbial N removal in eutrophic freshwater ecosystems through analyzing a typical shrimp aquaculture pond, which is located at Maoming (21° 32′ 55′′ N, 111° 22′ 46′′ E) (Fig. 1a). We collected the surface sediments (0–10 cm), with five replicates, from the water-land ecotone (WE) and the water-body zone (WZ), respectively (Fig. 1a). The sampling performed after the cultured shrimps were caught in August 2021, when the pond had a highly eutrophic status. The samples were collected using a gravity sampler. Sediments for molecular analysis were immediately placed in dry ice, transported to the laboratory and stored at -80 °C until nucleic acids extraction. Sediments for enrichment experiments were transported back to the laboratory using anaerobic bottles, then immediately transferred to the bioreactor. An additional sub-set of sediments was stored at 4 °C for measuring physicochemical properties. To gain a better understanding of microbial N removal function and adaptability, we manipulated the bioreactor with stable NH_4_^+^ and NO_2_^−^ supply to simplify environmental effects (Fig. 1b). For further details regarding the bioreactor experiments, please refer to Additional file 1.

### Physicochemical analysis

To assess the impact of environmental factors on the microbial N removal, a comprehensive group of physicochemical properties was analyzed. Specifically, the in situ measurements of water dissolved oxygen (DO) and sediments pH were performed using a hand-held meter (Extech Instruments, A FLIR Company, USA). Ion chromatography meter (ICS-600, Thermo, USA) was used to measure the concentrations of NH_4_^+^, NO_2_^−^ and NO_3_^−^. In brief, sediments were dried, homogenized, and passed through a 2.0 mm sieve. Then, NH_4_^+^, NO_2_^−^ and NO_3_^−^ were extracted from 2 g of the sieved sediments by shaking for 1 h at room temperature with 10 mL of 1 mol/L KCl. The activities of nitrate reductase (Nar: NO_3_^−^ → NO_2_^−^), nitrite oxidoreductase (Nxr: NO_2_^−^ → NO_3_^−^), nitrite reductases (Nir: NO_2_^−^ → NO), and ammonia monooxygenase (Amo: NH_4_^+^ → NH_2_OH) were measured according to the protocol of an ELISA kit (Jiangsu Yilaisa Biotechnology Co., Ltd). To evaluate the contributions of different pathways to the N removal, we measured the rates of major N transformations. For further details please refer to Additional file 1.

### DNA/RNA extraction and DNA-based quantitative PCR analysis

The DNA was extracted using a Power Soil DNA Isolation Kit (Mo Bio Laboratories, Carlsbad, CA, USA) with a grinding extraction method (Zhang et al. 2022). Only the DNA with an A_260/280_ ratio around 1.8 and an A_260/230_ ratio above 2.0 was kept for subsequent analysis. Microbial RNA was extracted using the RNeasy PowerSoil Total RNA Kit (Mo Bio Laboratories, Carlsbad, CA, USA) according to the manufacturers’ protocols. The quality of extracted RNA was checked by using NanoDrop (Thermo Sci-entific, USA) and agarose gel electrophoresis according to the methods described previously (Wang et al. 2019).

To quantify the anammox bacteria, we measured the copies of *hzsB* gene (Table S1) using real-time quantitative polymerase chain reactions (qPCR) (Miao et al. 2018). The DNA concentration was determined by the fluorescent method with Qubit 4 (Fluorometer, Thermo Scientific, USA). The plasmids DNA was amplified, cloned, extracted, and purified to construct a standard curve with SYBR Green method. Then the samples were analyzed using similarly methods and quantified according to the constructed standard curve. Each amplification was performed in a 20 μL reaction system, which included 10 μL of SYBR Green mix, 0.25 μL of primer (5 mM), 2 μL of template DNA (10 ng), and 7.75 μL of sterile deionized water. Only the qPCR amplifications with slopes between 3.39 and 3.92, amplification efficiency greater than 95%, and those showed a single dissolution peak were kept for final quantification.

### Metagenome and metatranscriptome sequencing analysis

To give a comprehensive profile of N removal related genes, shotgun metagenome sequencing was performed for the in situ sediments (i.e., WE and WZ) and samples collected from the reactor at 153 and 317 days. With the NEXTFLEX Rapid DNA-Seq Kit (Bioo Scientific, USA), DNA fragment libraries were constructed using 1 μg of high-quality DNA, and then sequenced on a Novaseq6000 platform at the Shanghai Majorbio Bio-pharm Biotechnology Co., Ltd (Shanghai, China). The raw data was filtered to remove reads with average quality scores < 20 using Trimmomatic v0.38, and the artificial duplicate reads were also removed (Bolger et al. 2014). The high-quality raw reads were then trimmed and assembled into contigs using MEGAHIT (v1.2.9), and only contigs ≥ 500 bp were kept for subsequent analysis. MetaGene was used to predict the open reading frames (ORFs) of all assembled contigs and translated into amino acid sequences. Then, KEGG database and NCycDB database were searched using DIAMOND blastx to identify the putative protein-coding sequences (Tu et al. 2019). Trans Per Million (TPM) values were used to determine significant difference among groups (Zhang et al. 2023). Contigs from different samples were binned using Metabat2 (v2.12.1) and MaxBin2 (v2.2.5). The original bins were consolidated and improved with Bin_refinement and Reassemble_bins module in metaWRAP (Zhang et al. 2023). The metagenome-assembled genomes (MAGs) were assessed in terms of completeness and contamination using CheckM (v1.0.12), and only the MAGs with completeness > 90% and contamination < 5% were kept (Luo et al. 2021). To perform taxonomic annotation, the ORFs of each gene were extracted and searched against the KEGG database and NCycDB database using DIAMOND blastx with an *e*-value ≤ 10^–5^. Genes annotated by KEGG were further classified as KOs, pathways, and modules.

To give a more accurate depiction of N removal function, the in situ sediments (i.e., WE and WZ) were selected for shotgun metatranscriptome sequencing (reactor samples failed in RNA extraction were not included). We used the Ribo-Zero rRNA removal kit (Illumina, USA) to remove rRNA, and then synthesized the cDNAs for sequencing on a Novaseq6000 platform at Shanghai Majorbio Bio-pharm Biotechnology Co., Ltd (Shanghai, China). We got approximately 15 Gb data for each sample. To remove non-coding RNA sequences, we employed multiple rRNA databases (e.g., SILVA, NCBI RefSeq databases) to filter the data. We predicted ORFs from the co-assembled contigs using Prodigal (v2.6.3), and annotated the predicted protein sequences using KEGG GhostKOALA. The per kilobase of exon model per million mapped reads (FPKM) values were calculated by mapping non-rRNA reads to all predicted ORFs. The GFOLD (v1.1.4) was used to identify differentially expressed genes (DEGs) (Cui et al. 2020). To reduce potential noise from marginally responsive genes, we applied a DEG filtering cutoff of 0.01 (− sc 0.01) and a |GFOLD value|> 1 (Yang et al. 2017).

### Statistical analysis

Linear regression analysis and scatter plot were used to assess correlations between any two parameters using the *glmnet* package in R. The ANOVA and *t*-test were performed to assess the difference using SPSS (version 24.0), and the significance level was examined at *p* < 0.05 (Zhang et al. 2022). The dissimilarities of between microbial communities were visualized using principal co-ordinates analysis (PCoA) with Bray–Curtis distances. We also conducted principal component analysis (PCA) to investigate the relationship between changes in metabolic pathways and reactor performance. Permutational multivariate analysis of variance (PERMANOVA) was performed to compare different sites and enrichment stages using the *VEGAN* package in R (version 3.4.4) (Dixon, 2003). The β-nearest taxon index (βNTI), which calculated by the *picante* R package, was used to infer the community assembly processes. To identify putative bacterial bioindicators, linear discriminant analysis (LDA) and effect size (LEfSe) analyses were performed (Segata et al. 2011). Spearman coefficient was used to measure the correlations between the environmental factors and dominant taxa. R packages including *corrplot* and *pheatmap* were used to perform the spearman analyses. Mantel text was used to determine the correlations between microbial communities and physicochemical factors as described previously (Liu et al. 2020). The co-occurrence networks were constructed by a phylogenetic Molecular Ecological Network Analysis (MENAP) pipeline (http://ieg4.rccc.ou.edu/mena) using Random Matrix Theory (RMT)-based methods according to a previous study by Zhang et al., (2023). Networks were visualized by the Gephi software. Structural equation modeling analysis (SEM), a powerful statistical method to reveal interactive relationships among manifest variables and latent variables, was used to analyze the relationships between N removal microorganisms and environmental factors. The SEM model with latent variable, manifest variables and path diagram was constructed using *plspm* package in R (Luo et al. 2021). Variation partition analysis (VPA) was used to quantify the relative contributions of the sediment physicochemical factors to microbial communities with the *vegan* package (Wu et al. 2019b). The niche width analysis was conducted using the *niche.width* function in *spaa* package in R (Jiao et al. 2020).

## Results

### Physicochemical characteristics

**Fig. 2:**
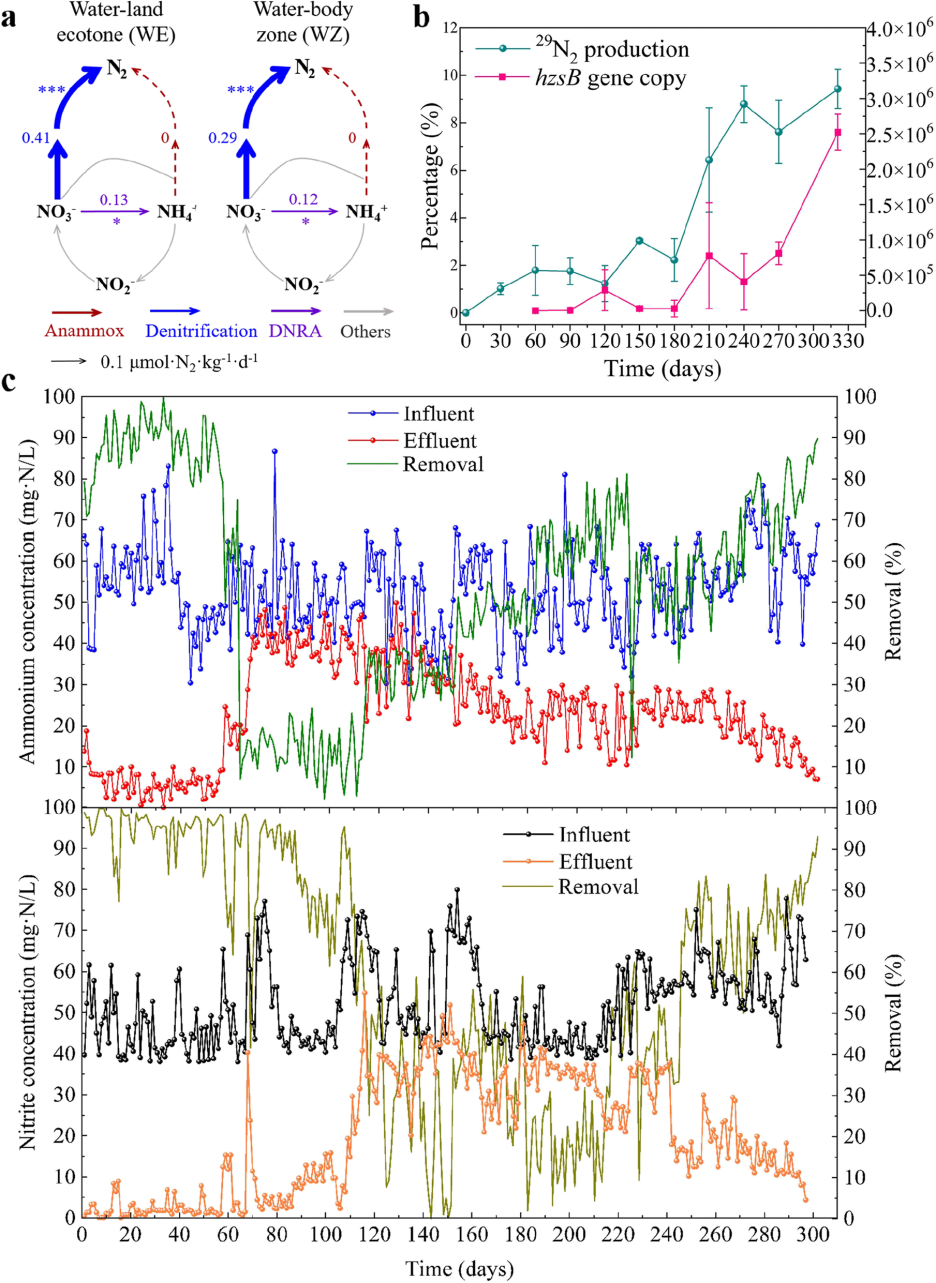
Nitrogen (N) removal characteristics of the in situ communities and those after the enrichment. **a** N removal rates determined by ^15^NO_3_^−^ isotope analysis of the in situ sediments (* *p* < 0.05; ** *p* < 0.01; *** *p* < 0.001). The dashed lines indicate undetected values. DNRA-dissimilatory nitrate reduction to ammonium. **b** Quantification of the anammox across the enrichment. **c+** N conversion and removal efficiency during the reactor enrichment with in situ sediments

To explore how the N-related nutrients affect the microbial N removal processes, we measured the NO_3_^−^, NO_2_^−^ and NH_4_^+^ of sediments collected in situ. The NH_4_^+^ and NO_3_^−^ were significantly (*p* < 0.05) lower in WE than that of WZ (Table S2), but the NO_2_^−^ in WE and WZ was undetectable. The DO in water was considerably lower in WE than that in WZ. The enzyme activity of Nxr in WE was significantly higher than that of WZ (Table S2), but the activity of Amo was much lower in WE than that of WZ (Table S2). The denitrification rate in WE was significantly higher than that of WZ (Fig. 2a), and a significant difference was also observed for the DNRA rate (*p* < 0.05). The differences of N-related nutrients and enzyme activities of in situ sediments suggested that the N transformation processes in WE and WZ might be different.

To verify environmental influences on the N removal processes, we performed reactor enrichment with a stable supply of NH_4_^+^ and NO_2_^−^. We found that the NH_4_^+^ removal rate was relatively high at the state of 1–57 days, then the removal of NH_4_^+^ and NO_2_^−^ decreased from 58 to 152 days (Fig. 2c). The inoculated bacteria were able to adapt the reactor conditions after 152 days incubation, which was recorded as bioreactor transition (BT). Then, an obvious increase of NO_2_^−^ removal was observed at the stage of 153–317 days, thus recorded as bioreactor enrichment (BE) of anammox bacteria. Isotopic analysis showed that the ^29^N_2_ produced by anammox bacteria increased from 0 to 9.41% during the enrichment (Fig. 2b). Correspondingly, the quantity of *hzsB* gene copies (Fig. 2b) and the anammox activity increased considerably across the enrichment. The enrichment experiments confirmed substantial N removal potentials of sediment microorganisms.

### Bacterial diversity and interactions

**Fig. 3:**
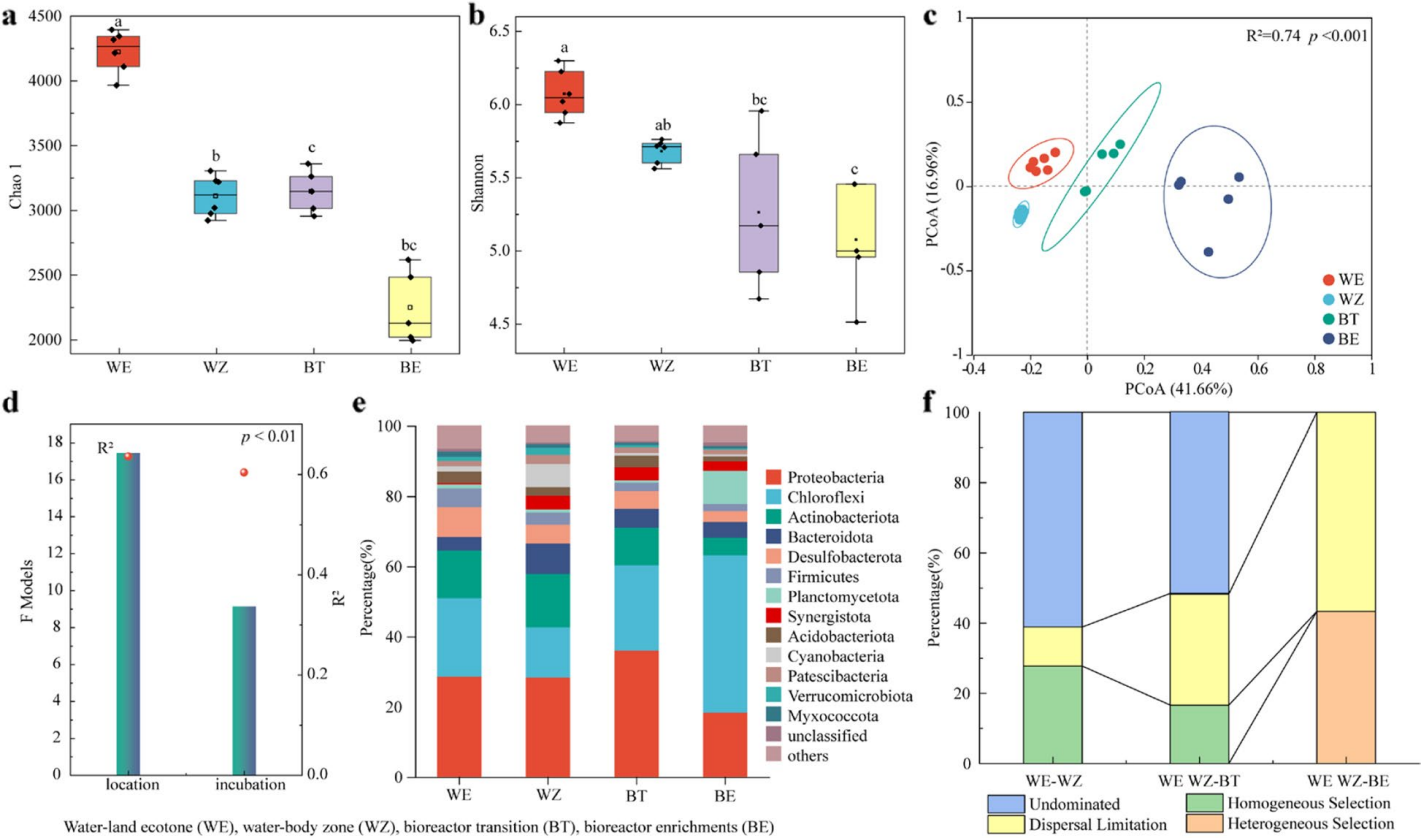
The diversity and composition of bacterial communities. **a** Chao1 and **b** Shannon indices showing the alpha-diversity of bacterial communities. Different lowercase letters above the bars indicated significant differences (*p* < 0.05) among groups. **c** Bray–Curtis distance-based PCoA showing the similarity of bacterial communities. **d** Permutational multivariate analysis of variance (PERMANOVA) analysis within in situ sampling locations and enrichment stages. The R-squared (R^2^) value represents the explanatory power and indicated by the red dots. **e** The relative abundance of major phyla based on the sequenced 16S rRNA gene. **f** The quantified major ecological processes governing the bacterial communities

To understand the microbial diversity and interactions, the 16S rRNA gene was sequenced. The Chao1 and Shannon indices showed significant differences (*p* < 0.01) between WE and WZ, and the α-diversity decreased across the enrichment (Fig. 3a, b). The Bray–Curtis based PCoA revealed distinct bacterial community structures (Fig. 3c), and the bacterial communities were significantly (PERMANOVA, *p* < 0.05) different among the in situ and enriched sediments (Fig. 3d). Similarly, the relative abundances of major phyla and genera (Fig. 3e and Fig. S1) were significantly different (*p* < 0.05). For example, Planctomycetota, Chloroflexi, Gemmatimonadetes and Synergistota were significantly enriched (*p* < 0.05). The contributions of different processes governing the bacterial communities at BT stage were similar to the in situ communities (Fig. 3f). However, the communities at stage BE were mainly governed by ecological niche-based dispersal limitation and heterogeneous selection.

The interactions among bacterial OTUs were visualized by network analysis (Fig. S2). The networks of in situ (WE and WZ) communities showed lower complexity and modularity (1.19 and 1.42) than those of BT and BE (Table S3). The large modules (≥ 10 nodes), which explained more than 50% of the network variations, showed that the network complex increased with the enrichment (Tables S3) under a stable condition. Specifically, the large modules increased to account for 50% after the enrichment due to the significant portion of microbial interactions and connections within the network. Moreover, there were many more negative interactions for in situ community networks than those of enriched community networks, and the positive interactions increased across the enrichment.

### N removal potentials, functional expression and pathways

**Fig. 4:**
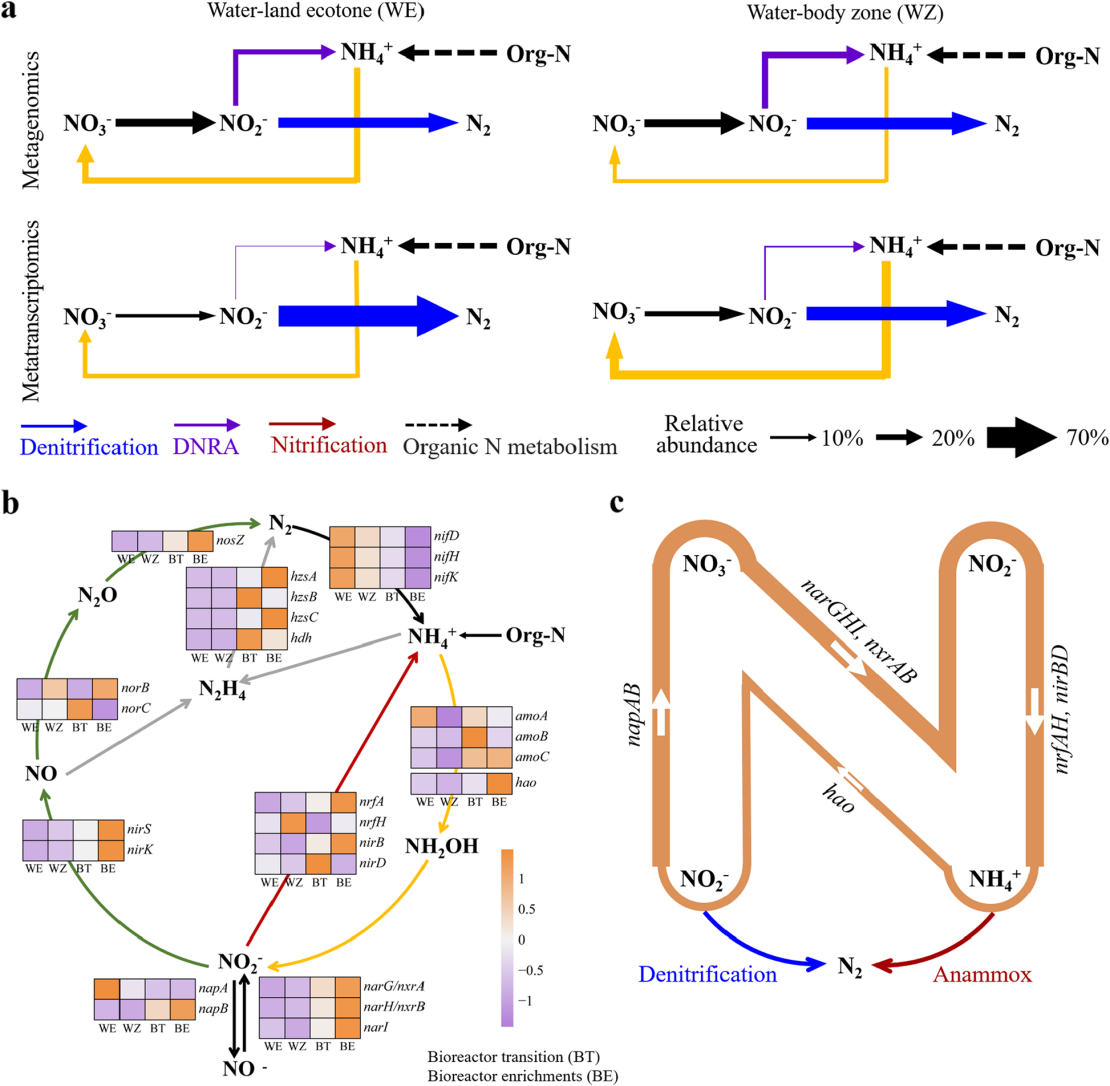
The abundance and expression the nitrogen (N) genes in metabolic pathways. **a** The overall gene abundance and expression of the N-related pathways of in situ communities. The total abundance was calculated by summing the transcripts per million (TPM) values of all functional genes, and then calculating the relative abundance of each gene to the total abundance. DNRA-dissimilatory nitrate reduction to ammonium. **b** The abundances of genes involved in N metabolism compared before and after the enrichment. Genes abundances were normalized into TPM counts. **c** Proposed pathways for NO_2_^−^ and NH_4_^+^ loop oxidation and reduction during the enrichment in the reactor. The thickness of white arrows represents the relative abundance of the related genes

To understand the microbial function involved in N removal pathways, we explored the abundance and expression of the related genes (Fig. 4a). We found that the high gene abundance not necessarily related to high expression. Specifically, the DNRA genes (*nrfA/H*) showed relatively high abundance but low expression of in situ communities. On the contrary, a relatively low abundance of denitrifying genes (*norB/C, nirS/K*) with relatively low abundance were highly expressed in WE, while nitrifying genes showed high expression in WZ. Also, the gene expression, especially of key denitrifying genes (e.g., *nosZ* and *nirS*) (Fig. S3a), was significantly different between WE and WZ. However, the richness of gene and function in WE was significantly higher than that of WZ (Fig. S4).

The metagenome sequencing indicated that the denitrification and DNRA were the major N removal pathways across the enrichment (Fig. 4b, Fig. S3b). The anammox bacteria were only significantly enriched at the BE stage (Fig. S3b). Specifically, the genes in responsible for NO_2_^−^$\leftrightarrow$ NO_3_^−^ (*narG, narI, narH*) showed the highest abundance among all the N removal genes (Fig. 4b). All subunits of the hydrazine synthase (HZS) also showed abundance (Fig. 4b). Notably, the abundance of *nrfA/H* genes at the BE stage was significantly higher than those before enrichment (Fig. 4b), indicating an active reduction of NO_2_^−^ to NH_4_^+^. The ammonium oxidation gene (*hao*) also showed significantly higher abundance (> 6%) after the enrichment of 317 days (BE) (Fig. 4b). Overall, the denitrification, DNRA and anammox were the major processes to remove the NO_2_^−^ and NH_4_^+^ in the bioreactor system (Fig. 4c). Pearson's correlation was used to reveal the relationship among DNRA, anammox and denitrifying genes before and after enrichment (Fig. S5). Comparative analysis with before the enrichment conditions indicated a tighter relationship among denitrification, DNRA, and anammox after the enrichment. Mantel tests indicated that most of bacterial phyla were significantly (*p* < 0.01) correlated with various metabolic pathways (Fig. S6).

### Metabolic potentials and their relations to microbial community

**Fig. 5:**
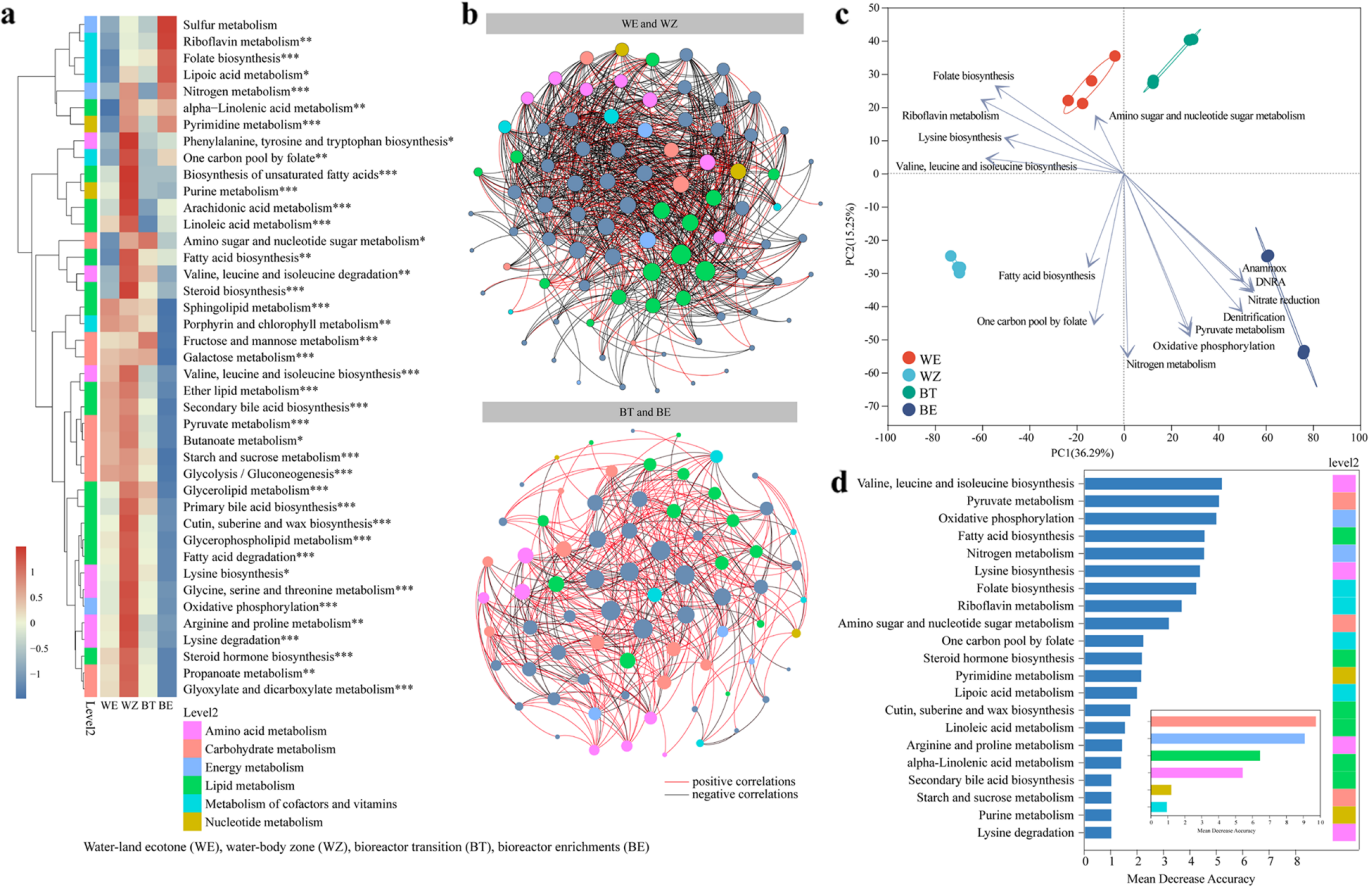
Relationships between microbial metabolic pathways and community composition. **a** The abundance of KEGG modules calculated by homogenization and normalized into transcripts per million (TPM) counts (* *p* < 0.05; ** *p* < 0.01; *** *p* < 0.001). **b** The co-occurrence networks of metabolic pathways with the main microbial phyla. The node size represents the degree of each pathway or phylum. The black and red links represent negative and positive correlations, respectively. **c** PCA plots showing the variations of the top 10 metabolic pathways (determined by random forest) in relation to the nitrogen (N) removal processes (DNRA-dissimilatory nitrate reduction to ammonium, anammox, nitrate reduction and denitrification). **d** The identified important metabolic pathways for predicting the contribution of the key metabolisms

The quantified KEGG metabolic annotation (level 3) showed more complex pathways of in the in situ communities than that in the enriched communities (Fig. 5a). The bipartite networks of microbial communities and metabolic pathways exhibited a higher proportion of negative correlations than positive correlations (Fig. 5b) of in situ communities, indicating clear competition or inhibition. However, the positive connections increased considerably after the enrichment, indicating an enhance of cooperation across the enrichment (Fig. 5b). Moreover, the processes of DNRA, anammox, nitrate reduction and denitrification showed significant positive correlations on to the top 10 metabolic pathways at the BE stage (Fig. 5c). Additionally, nutrients (e.g., vitamins and lipid) also significantly affected the bacterial composition of in situ communities. The random forest analysis indicated that the carbohydrate metabolism, energy metabolism and amino acid metabolism would be the major contributors to predict the microbial N removal function (Fig. 5d). Based on the KEGG analysis results, carbohydrate metabolism and amino acid metabolism are the pathways with the highest proportions, while pathways with significant differences include carbohydrate metabolism, amino acid metabolism, and energy metabolism (Fig. S7). The carbohydrate metabolism of glycolysis and the pentose phosphate pathway showed notable variations, and the biosynthesis pathways of amino acid (e.g., lysine and arginine) also exhibited significant differences. Energy metabolism was significantly different in the TCA cycle and oxidative phosphorylation pathways. These specific pathway differences are further detailed in the subsequent sections. According to the COG analysis results, functional modules such as carbohydrate transport and metabolism, as well as energy production and conversion, have the highest proportions and exhibit significant differences (Fig. S7). The partial Mantel tests confirmed that the energy metabolism and carbohydrate metabolism were significantly correlated with the N removal function (Fig. S8).

### Relationships between N removal capacities and environmental factors

**Fig. 6:**
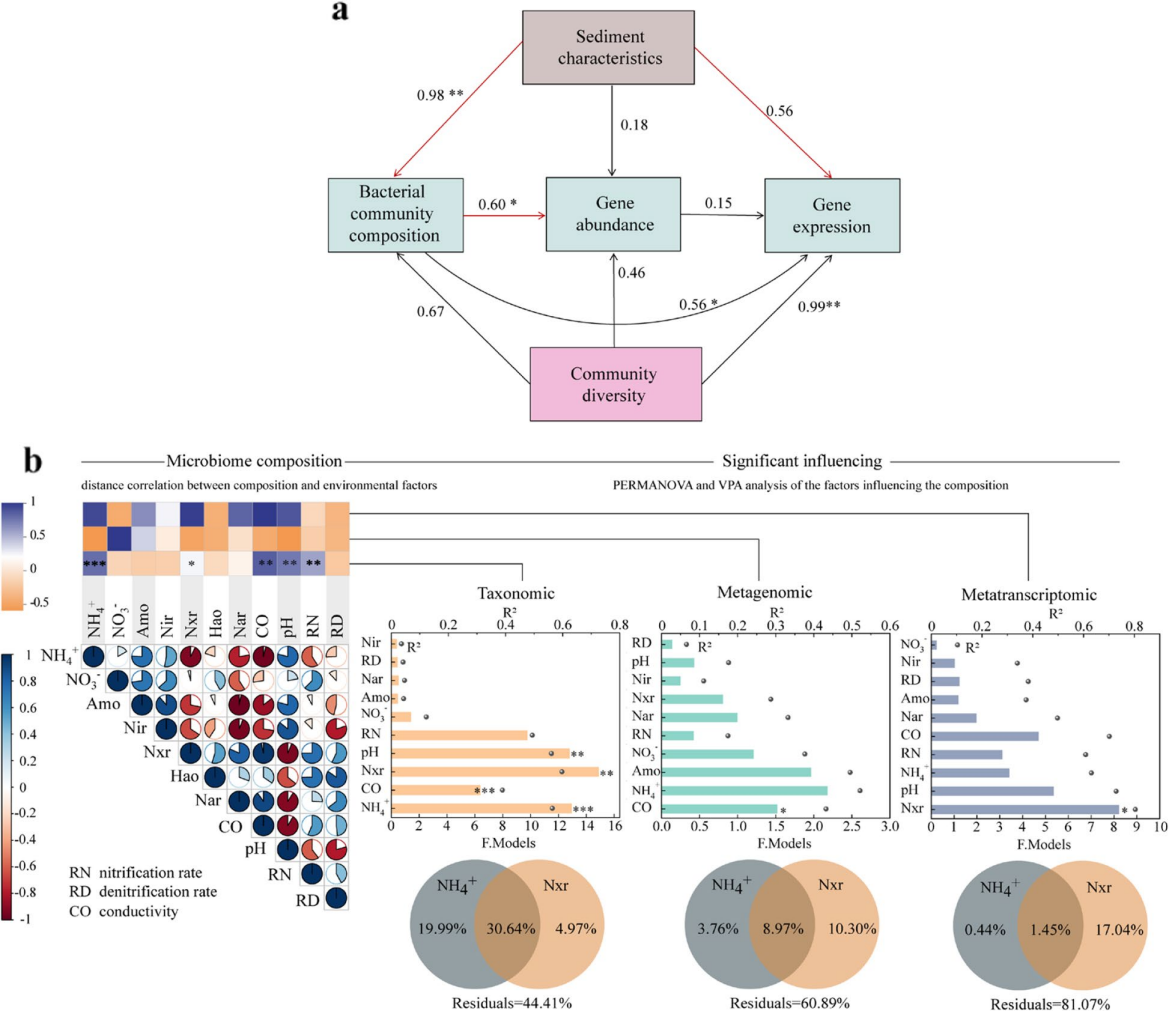
Relationships between abiotic and biotic factors. **a** Path diagrams estimating the effects of abiotic and biotic factors on the abundance and expression of nitrogen (N) genes. Black and red lines represent positive and negative effects, respectively. Numbers adjacent to the arrows are standardized path coefficients. Single headed arrows refer to unidirectional causal relationships (* *p* < 0.05; ** *p* < 0.01). **b** Integrated analysis of relationships between environmental factors, bacterial communities, and N removal processes. Pairwise Spearman’s correlation matrix of the environmental factors showed by the pie charts, and Mantel tests were used to determine the relationships between each pair of abiotic and biotic matrix. Permutational multivariate analysis of variance (PERMANOVA) screened statistically significant factors and variance partitioning analysis (VPA) showed the effects of major factors on microbial communities. The R-squared (R^2^) value represents the explanatory power of each factor on sample variations

The SEM showed that the measured abiotic factors (Table S2) only significantly correlated with the community composition (path coefficients = -0.98, *p* < 0.01) (Fig. 6a), but showed no significant relationships with the abundance and expression of N removal genes (*p* > 0.05) (Fig. S9). However, the community composition was significantly correlated with the N removal genes’ abundance and expression (Fig. 6a). Pairwise Spearman’s correlation and partial Mantel tests confirmed that the abundance and expression of the N removal genes were not significantly (*p* > 0.05) correlated with abiotic factors (Fig. 6b). Bacterial community composition presented strong positive correlations with the content of NH_4_^+^, as well as the activities of Nir and Nxr enzymes (Fig. 6b). The conductivity and pH also could significantly influence the bacterial community composition (Fig. 6b). In addition, the NH_4_^+^ and Nxr showed significant correlations with the Shannon and Chao1 index of the bacterial community (*p* < 0.01) (Fig. S10). The VPA indicated that NH_4_^+^ and Nxr could explain much higher variations of bacterial community composition than that of genes abundance and expression (Fig. 6b).

### Molecular metabolisms of N removal microorganisms

To clarify the molecular mechanism regulating microbial N removal, 71 and 83 MAGs carrying N removal genes were screened from lake sediments and enrichment bioreactor, respectively (Fig. S11). Before enrichment, two MAGs (WE1_MAG31/W_N1 and WE2_MAG66/ W_N2) encompass nearly all essential genes for denitrification and DNRA except for those responsible for the conversion of N_2_O to N_2_. After enrichment, two MAGs (B_MAG92/B_SDN and B_MAG181/B_DNR) carry all the genes required for DNRA (i.e., *nrfA, nirB*). Also, B_SDN encompasses nearly all the required genes involved in denitrification. B_MAG74 (B_AMX), which classified as *Candidatus* Jettenia and showed the highest abundance, carries all the functional genes for anammox, denitrification and DNRA. However, the functional genes of B_SDN and B_DNR exhibited a distinct distribution compared to those after enrichment (Fig. S11). Moreover, the majority of MAGs do not cluster within the same subgroups in the MAG-based phylogenetic tree before and after the enrichment, indicating adaptive evolution in response to the new environmental conditions (Fig. S12).

**Fig. 7:**
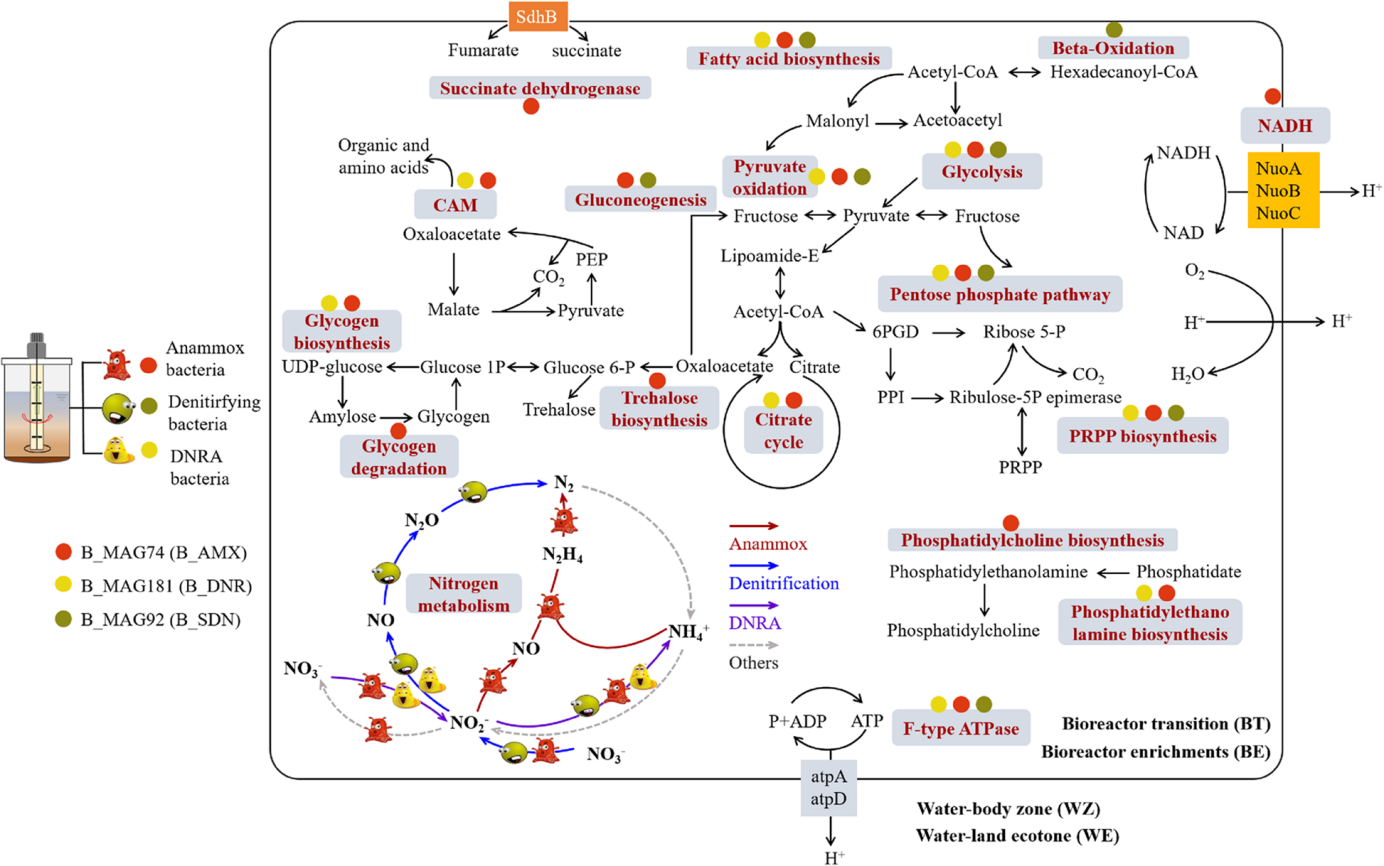
Potential metabolism pathways of nitrogen (N) removal coupled by representative metagenome-assembled genomes (MAGs) with the highest abundance and quality in each process (B_SDN, MAG92; B_DNR, MAG181; B_AMX, MAG74). Metabolic potential of MAGs inferred from BlastKOALA. CAM: crassulacean acid metabolism. DNRA-dissimilatory nitrate reduction to ammonium

**Fig. 8:**
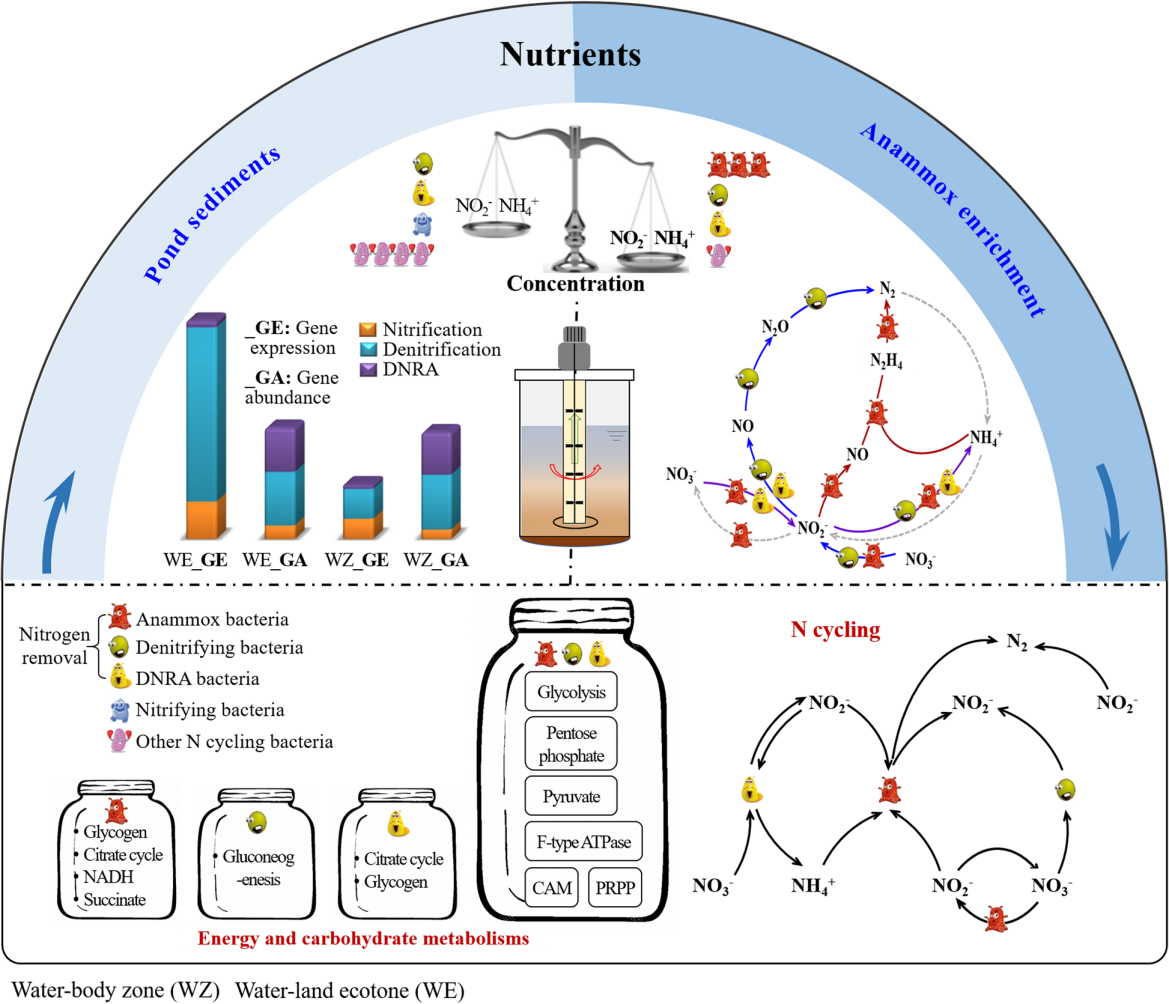
A conceptual model illustrating the mechanisms underlying the diversity, interaction and adaptation strategies of microorganisms involved in dissimilatory nitrate reduction to ammonium (DNRA), anammox and denitrification

Subsequently, we constructed their potential metabolic pathways (Fig. 8 and Fig. S13). Compared to before enrichment conditions, B_AMX, B_SDN, and B_DNR showed higher completeness and richness in carbohydrate metabolism (Fig. 7 and Fig. S13). The energy metabolism processes of the enriched MAGs became more complete (Fig. 7 and Fig. S13). We identified complete carbohydrate metabolism pathways of inorganic and organic respiration (e.g., glycolysis, pyruvate oxidation, pentose phosphate pathway, and PRPP biosynthesis) in the B_SDN, B_AMX, and B_DNR (Fig. 7). Notably, B_AMX exhibited unique carbohydrate metabolism pathways such as glycogen degradation and trehalose biosynthesis, differentiating it from the other MAGs. In terms of energy metabolism, F-type ATPase emerged as a common pathway among B_SDN, B_AMX, and B_DNR, indicating a fundamental similarity in energy production (Fig. 7). Specifically, B_AMX uniquely featured energy metabolism pathways involving NADH and Succinate dehydrogenase. Additionally, we also successfully identified other complete metabolic pathways encompassing lipid metabolism, nucleotide metabolism, and other critical cellular processes (Table S4). Subsequently, in the exploration of amino acid metabolism and metabolism of cofactors and vitamins, we pinpointed unique or specific amino acids and cofactors/vitamins within the MAGs derived from the enrichment of B_SDN, B_AMX, and B_DNR (Table S4).

## Discussion

The denitrification, DNRA and anammox, which contribute significantly to the N removal, could be regulated to manage the N status in eutrophic ecosystems (Wang et al. 2020). However, the biotic and abiotic mechanisms controlling the function of N removal microorganisms remain unclear (Ma et al. 2020). Due to high impacts and complex interactions of bacterial community on the N metabolism (Wang and Li 2023), a stable and simple system is especially necessary to verify the N removal mechanisms in natural ecosystems (Widder et al. 2016). By establishing a reactor system to enhance N removal, this study revealed the influences and underlying mechanisms of variations in bacterial community composition, as well as the N removal potentials and their functional expression. We found that the N removal capability of in situ communities in the eutrophic ecosystem is largely inhibited. However, the microorganisms in the reactor sediments showed great N removal potentials by coupling the denitrification, DNRA and anammox. Notably, the anammox bacteria showed strong adaptability to the given conditions during the enrichment, as evidenced by their significant increase determined by the qPCR.

Previous studies indicated that microbial communities and their functions could be affected by various environmental factors and biological interactions (Liu et al. 2018; Ma et al. 2020). Moreover, the abundant bacteria often exhibit much higher nutrient utilization capabilities than the rare taxa (Zhang et al. 2022). The microbial diversity and metabolic pathways are good reflections of nutrients utilization and adaptation to the environments (Xing et al. 2021; Wagg et al. 2019). Our results only showed strong environmental effects on the bacterial composition rather than the gene abundance and expression. However, we speculate that the N-availability may influence the abundance and expression of N removal genes (Séneca et al. 2021). For example, the N-rich conditions would result in a relatively low expression of N removal genes due to the high availability of environmental N. High bacterial diversity always results in diverse metabolic pathways, thus lead to significant differences in N metabolism of different in situ communities. So, the in situ communities showed more complex metabolic pathways than that of the enriched communities.

The interaction between denitrification, DNRA and anammox is another key factor regulating the N removal. We found that the network modularity and positive connections of bacterial communities increased considerably after the enrichment. The positive correlations among taxa reflect ecological or functional similarity (Zeng et al. 2021) due to environmental filtering, while taxa with divergent niches always negatively correlated (Pandey et al. 2023). Thus, the wider ecological niches and stronger competition of in situ communities indicated complex functions involved in the N transformation (Jiao et al. 2020). The bipartite network between community composition and metabolic pathways also confirmed clear competition among in situ microbial communities. This could be due to microbial competition for nutrients (Yang et al. 2022; Yuan et al. 2021), which confirmed by the strong correlation between nutrient substances and microbial composition. Additionally, some microbial taxa can use intermediate metabolites produced by other microbes (Cheng et al. 2022). This cross-feeding can increase the competition and overall complexity of microbial ecosystem.

The enrichment always results in simple microbial community with relatively low diversity (Miao et al. 2023) to study the responses of specific taxa to the targeted factors, but investigating these issues at an appropriate scale still remains challenging (Zheng et al. 2016). We found that the positive network correlations increased across the enrichment, suggesting an increase of cooperative relationships. The observed significant relationship among the functional genes of denitrification, DNRA, and anammox after enrichment implies a coordinated response of these N cycling pathways within the microbial community. This coherence suggests a potential synergistic interaction among different microbial groups, supporting the concept of mutualistic relationships (Zhang et al. 2023). The intricate interplay among these functional genes highlights the importance of microbial cooperation in N cycling processes. Our results also indicated an enhanced N removal across the enrichment through the microbial interactions. The enrichment effectively unleashes the metabolic potentials of specific pathways (Banerjee et al. 2016) such as the energy and carbohydrate metabolisms observed herein. Previous study also suggested that enrichment could increase the co-occurrence network modularity because interconnected nodes within modules share analogous functions (Wang et al. 2021a). A relatively high environmental stress could result in more positive connections than negative interactions (Hernandez et al. 2021). Thus, the N removal microorganisms coexisted herein may be partly due to their cooperative interactions to share similar niches (Wang et al. 2021b). Overall, the strong determinism due to the robust environmental filtering and biotic interactions is a major factor affecting the community assembly during our enrichment (Liu et al. 2022; Zhou and Ning 2017).

Microbial communities possess a broad adaptive capacity, enabling them to respond to different environmental conditions and fulfill their functional roles (Wani et al. 2022). We found that the decrease of microbial diversity under a consistent supply of NH_4_^+^ and NO_2_^−^ enhanced the N removal capacity, particularly through the anammox pathway (Tanimu et al. 2015). The consistent supply of anammox substrates during the enrichment might relax selection pressures on the N removal microorganisms, resulting in low interconnectedness and high ecological equivalence. The observed convergence in the phylogenetic of denitrification processes further underscores the adaptability of microbial communities to specific environmental niches. The results of the phylogenetic tree analysis indicate a high degree of similarity in the phylogenetic of removal N processes, including denitrification, DNRA, and anammox. This finding aligns with the broader theme of microbial adaptability explored in this study. Essentially, the specific conditions established in the enrichment system could specify individual contributions of microbial entities to the overall N removal (Liu et al. 2021). Interestingly, we found that the microbial-mediated N removal was dominated by the conversion of NO_2_^−^, including the pathways of NO_2_^−^ → N_2_ and NO_2_^−^$\leftrightarrow$ NO_3_^−^ (Zhou et al. 2023). The reciprocal conversion between NO_2_^−^ and NO_3_^−^ are intricately interconnected through the reactions that consume NO_2_^−^, and the transformation of NH_4_^+^  → NO_2_^−^ also provides substrate NO_2_^−^ for N removal. Additionally, the DNRA process facilitates the conversion of NO_2_^−^ and NO_3_^−^ to NH_4_^+^, and supplies substrates for the anammox. Such NO_2_^−^ loop consequently could increase the denitrification and anammox (Chen et al. 2023; Zhou et al. 2023). Overall, the adaptability of microorganisms to the N-rich conditions highlighted significant potentials of N removal in eutrophic ecosystems. This study demonstrates significant interactions and adaptive strategies of N removal microorganisms in a simplified enriched community, which will increase our understanding of the robustness of N removal processes and the resilience of microbial communities in situ. However, we also should acknowledge the inherent differences between enrichment conditions and actual sediment environments. These differences may mean microbial activities in the enrichment system are somewhat different from that of natural ecosystems.

Despite the laboratory enrichment have significantly simplified the bacterial communities, interactions among microorganisms still remains complex (Zhou et al. 2021). Therefore, microorganisms in the enrichment system also showed diverse potentials to procure and utilize nutrients. Comparative analysis revealed noteworthy changes in carbohydrate metabolism after enrichment, indicating a fundamental similarity in energy generation and precursor molecule synthesis This shared utilization suggests a strategic adaptation for efficient resource utilization and energy conservation within the microbial community (Zhou et al. 2021). Moreover, the anammox MAG may support the other two types of MAGs under N-rich conditions (Junge et al. 2004). B_AMX employs the NADH and succinate dehydrogenase pathways uniquely, indicating a distinctive energy metabolism strategy that potentially enhances flexibility in adapting to varying environmental conditions (Wu et al. 2023). Additionally, its exclusive features in glycogen degradation and trehalose biosynthesis pathways further underscore a unique metabolic strategy, enhancing adaptability to specific environmental conditions (Wei et al. 2023). The presence of specific amino acids and cofactors/vitamins within B_SDN, B_AMX, and B_DNR implies a tailored metabolic strategy involving the production and exchange of specific metabolites (Yu et al. 2022). This investigation into shared and exclusive metabolic pathways suggests a cooperative relationship and adaptability among MAGs, while also underscoring potential interdependencies and the utilization of metabolites among these N removal processes (Dan et al. 2023). We also should acknowledge that laboratory enrichment cannot fully reflect the natural environments, where involving more variables and microbial interactions that may affect the diversity and function of N removal microorganisms.

## Conclusions

This study reveals the interactions and adaptive strategies of denitrification, DNRA and anammox communities in N removal by analyzing field samples and conducting laboratory enrichment experiments (Fig. 8). We found that microbial communities and the expression of denitrification, DNRA, and anammox genes are significantly influenced by NH_4_^+^ and Nxr, as well as biological interactions, particularly when subjected to environmental stress. Under enrichment conditions, the influence of in situ environmental factors is diminished, potentially fostering more positive connections among microbial communities through shared ecological niches, rather than competitive interactions. Additionally, the presence of shared metabolic features among B_SDN, B_AMX, and B_DNR suggests cooperative relationships and adaptability, while specific metabolic pathways imply different strategies for environmental adaptation. This shared and exclusive utilization of metabolic pathways underscores potential interdependencies and metabolite exchanges among N removal processes. Overall, this study holds important implications for managing N levels and associated eutrophication in freshwater ecosystems.

## Supplementary Information

Supplementary Material 1.

## Data Availability

The raw Illumina data sets used in this study have been deposited in the NCBI repository under accession numbers PRJNA906637, PRJNA954064, and PRJNA953160.
