# Publisher Correction: Mechanisms underlying the interactions and adaptability of nitrogen removal microorganisms in freshwater sediments

**DOI:** 10.1007/s44307-024-00029-5

**Published:** 2024-07-04

**Authors:** Dandan Zhang, Huang Yu, Xiaoli Yu, Yuchun Yang, Cheng Wang, Kun Wu, Mingyang Niu, Jianguo He, Zhili He, Qingyun Yan

**Affiliations:** 1 Marine Synthetic Ecology Research Center, Southern Marine Science and Engineering Guangdong Laboratory (Zhuhai), School of Environmental Science and Engineering/Life Sciences/Ecology, Guangdong Provincial Observation and Research Station for Marine Ranching in Lingdingyang Bay, China-ASEAN Belt and Road Joint Laboratory On Mariculture Technology, State Key Laboratory for Biocontrol, Sun Yat-Sen University, Zhuhai, 519082 China; 2 School of Resources Environment and Safety Engineering, Key Discipline Laboratory for National Defense for Biotechnology in Uranium Mining and Hydrometallurgy, University of South China, Hengyang, 421001 China

**Correction** **:** **Advanced Biotechnology 2, 21 (2024)**

**https://doi.org/10.1007/s44307-024-00028-6**

Following publication of the original article (Zhang et al. 2024), it was reported that there was an error in the author affiliation of the corresponding author due to a typesetting mistake. Dr. Qingyun Yan’s affiliation number is supposed to be 1 instead of 2.

The correct author affiliation has been provided in this Correction and the original article (Zhang et al. 2024) has been corrected.

## References

[CR1] Zhang D, Yu H, Yu X, et al. Mechanisms underlying the interactions and adaptability of nitrogen removal microorganisms in freshwater sediments. Adv Biotechnol. 2024;2:21. 10.1007/s44307-024-00028-6. 10.1007/s44307-024-00028-6 PMC11740870 39883300

