# Supplementary material for: Mechanisms underlying the interactions and adaptability of nitrogen removal microorganisms in freshwater sediments
Source: Adv Biotechnol (Singap). 2024 Jun 17;2(3):21. doi: 10.1007/s44307-024-00028-6 (PMC11740870; doi:10.1007/s44307-024-00028-6)
Supplement: Supplementary file 1 — Supplementary Material 1. [file 44307_2024_28_MOESM1_ESM.docx]

**Supplementary Information**

**Table S1** Corresponding programs of the used PCR primers.

| Gene | Primers | Sequence (5’-3’) | Thermal profile |
| --- | --- | --- | --- |
| 16S rRNA | 338F | ACTCCTACGGGAGGCAGCAG | 95 ℃ for 5 min; then 30 cycles of 95 ℃ for 30s, 53 ℃ for 45s, 72 ℃ for 60s; 72 °C for 5 min. |
|  | 806R | GGACTACHVGGGTWTCTAAT |  |
| *hzsB* | 396F | ARGGHTGGGGHAGYTGGAAG | 95 ℃ for 3 min; then 35 cycles of 95 ℃ for 30s, 56 ℃ for 30s, 72 ℃ for 40s; 72 °C for 5 min. |
|  | 742R | GTYCCHACRTCATGVGTCTG |  |

**Table S2** Biogeochemical parameter measured in the two sites.

| Samples | DO  (mg/L) | C/N (%) | pH | NO_3_^-^  (mg·N/kg) | NO_2_^-^  (mg·N/kg) | NH_4_^+^  (mg·N/kg) | TC  (mg/g) | TN  (mg/g) | SO_4_^2-^  (mg/kg) | Amo (U/g) | Nir (U/g) | Nxr (U/g) | Nar (U/g) |
| --- | --- | --- | --- | --- | --- | --- | --- | --- | --- | --- | --- | --- | --- |
| WE | 23.21±1.08^a^ | 9.88^a^ | 7.63 ± 0.1^a^ | 0.37 ± 0.03^b^ | 0.01^a^ | 126.39 ± 15.32^b^ | 20.89 ± 0.51^b^ | 3.44 ± 0.01^b^ | 0.91 ± 0.01^b^ | 2009.15 ± 297.14^b^ | 1708.73 ± 284.67^b^ | 1909.84 ± 61.46^a^ | 0.82 ± 0.05^a^ |
| WZ | 11.57±0.19^b^ | 8.98^b^ | 7.19 ± 0.1^b^ | 0.76 ± 0.26^a^ | 0^a^ | 629.81 ± 53.73^a^ | 51.85 ± 0.22^a^ | 5.01 ± 0.07^a^ | 1.03 ± 0.01^a^ | 2094.05 ± 174.38^a^ | 2017.12 ± 324.01^a^ | 1352.58 ± 178.31^b^ | 0.84 ± 0.01^a^ |

The data presented as mean ± standard deviation (n = 6). The different superscript letters mean statistical significance (*p* < 0.05).

Water-land ecotone (WE), water-body zone (WZ)

**Table S3** Topological parameters in bacterial co-occurrence networks

| Type | Average degree | Modularity | Density | Average clustering coefficient | Negative connectivity (%) | Positive connectivity (%) |
| --- | --- | --- | --- | --- | --- | --- |
| WE | 4.673 | 1.193 | 0.007 | 0.331 | 86.38 | 13.62 |
| WZ | 3.712 | 1.418 | 0.006 | 0.226 | 66.09 | 33.91 |
| BT | 9.56 | 2.192 | 0.023 | 0.664 | 38.09 | 61.91 |
| BE | 31.43 | 2.493 | 0.158 | 0.691 | 16.32 | 83.67 |

Water-land ecotone (WE), water-body zone (WZ), bioreactor transition (BT), bioreactor enrichments (BE)

**Table S4** Principal metagenome-assembled genomes (MAGs)include pathways for amino acid metabolism and cofactors and vitamins metabolism.

|  |  | B_AMX | B_DNR | B_SDN | W_N1 | W_N2 |
| --- | --- | --- | --- | --- | --- | --- |
| Amino acid metabolism | Glycine | + | + | + | + | + |
|  | Cysteine | + | + | + | + | + |
|  | Valine  isoleucine | + | + | - | + | + |
|  | Leucine | + | - | - | - | - |
|  | Ornithine | + | + | - | + | + |
|  | Arginine | + | + | - | + | + |
|  | Proline | + | + | + | + | + |
|  | Tryptophan | + | + | - | - | - |
|  | Lysine | - | - | - | + | + |
|  | Serine | - | - | - | + |  |
|  | Histidine | - | - | + | - |  |
|  | Threonine | - | - | - | - | + |
| Metabolism of cofactors and vitamins | Biotin biosynthesis | + | - | - | - | + |
|  | Lipoic acid biosynthesis | + | - | + | + | + |
|  | Heme biosynthesis | + | + | - | - | - |
|  | Menaquinone biosynthesis | + | + | + | - | - |
|  | C1-unit interconversion | - | - | - | + | + |
|  | NAD biosynthesis | - | - | - | + | + |
|  | Pantothenate biosynthesis | - | - | - | - | - |
|  | Lipoic acid biosynthesis | - | - | - | - | - |
|  | Coenzyme A biosynthesis, | - | - | + | - | - |
|  | Pantothenate biosynthesis | - | - | - | + | + |

+: Present; -: Absent

B_MAG74 (B_AMX), B_MAG181 (B_DNR), B_MAG92 (B_SDN), WE1_MAG31 (W_N1) and WE2_MAG66 (W_N2)

Water-land ecotone (WE), water-body zone (WZ), bioreactor transition (BT), bioreactor enrichments (BE)


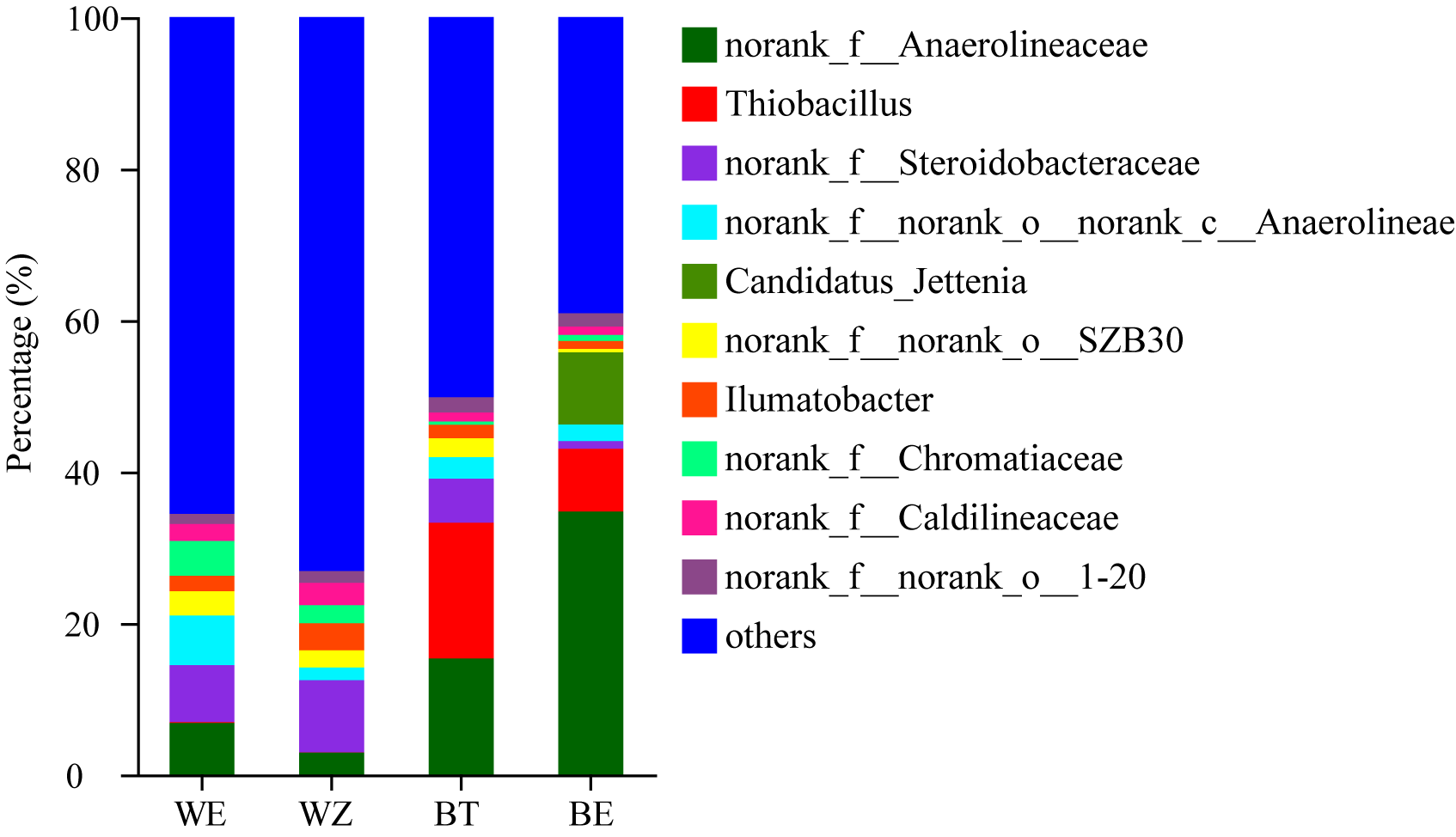


**Fig. S1** The relative abundance of major bacterial genera among the *in situ* and enriched sediments.

**
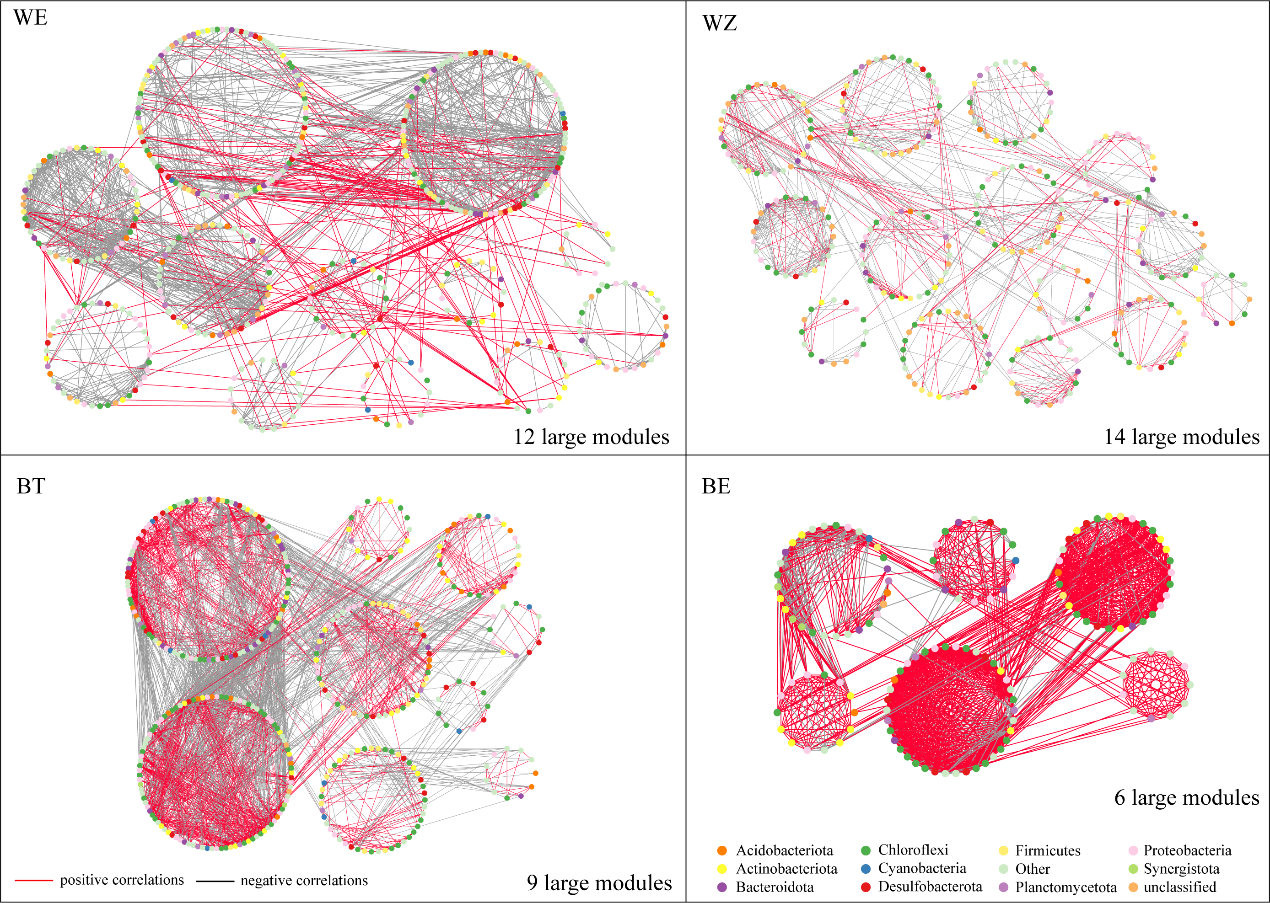
**

Water-land ecotone (WE), water-body zone (WZ), bioreactor transition (BT), bioreactor enrichments (BE)

**Fig. S2** Network of bacterial OTUs among different sites and stages. The red and black links represent positive and negative correlations, respectively.


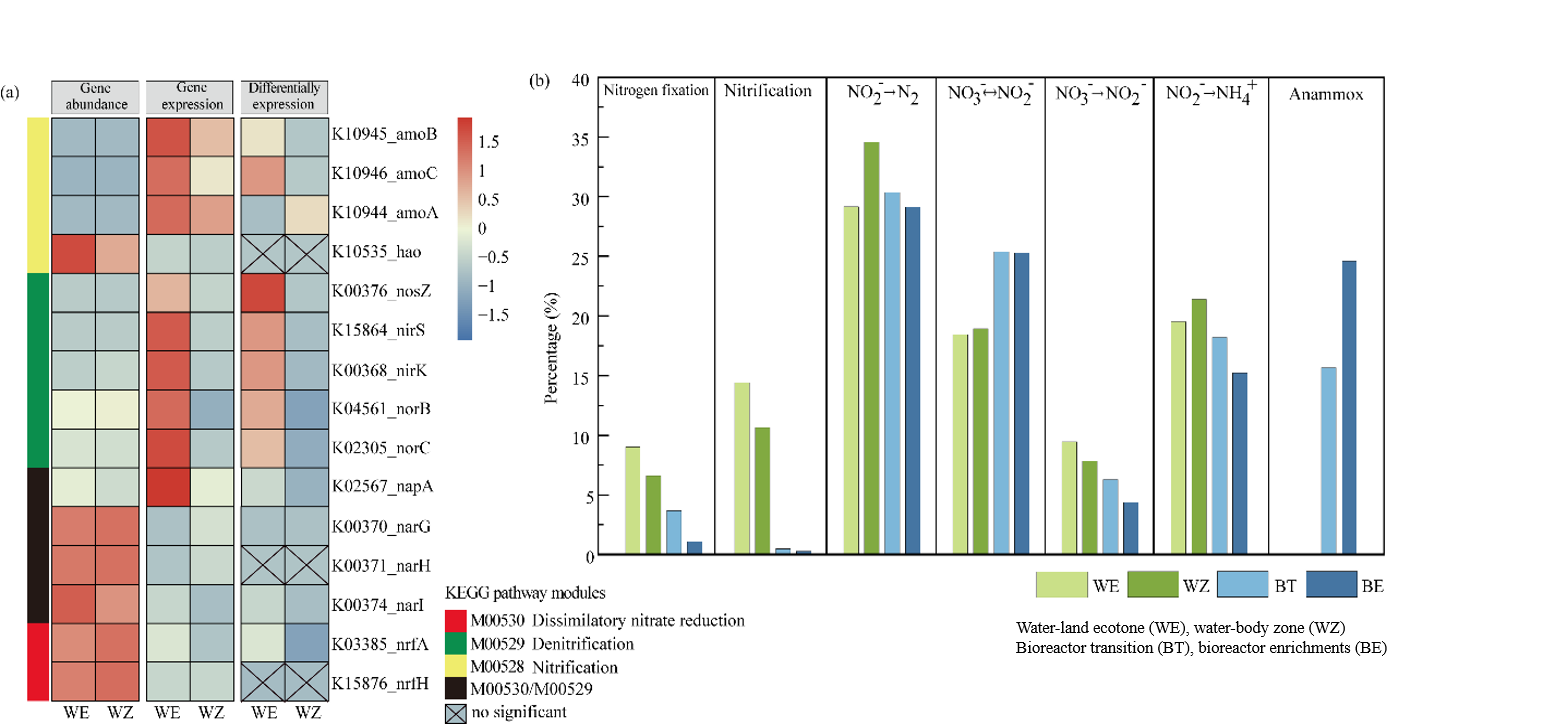


**Fig. S3** Differences in nitrogen (N) cycle gene abundance between the two sites (a) and nitrogen (N) metabolism pathways during reactor operation (b).


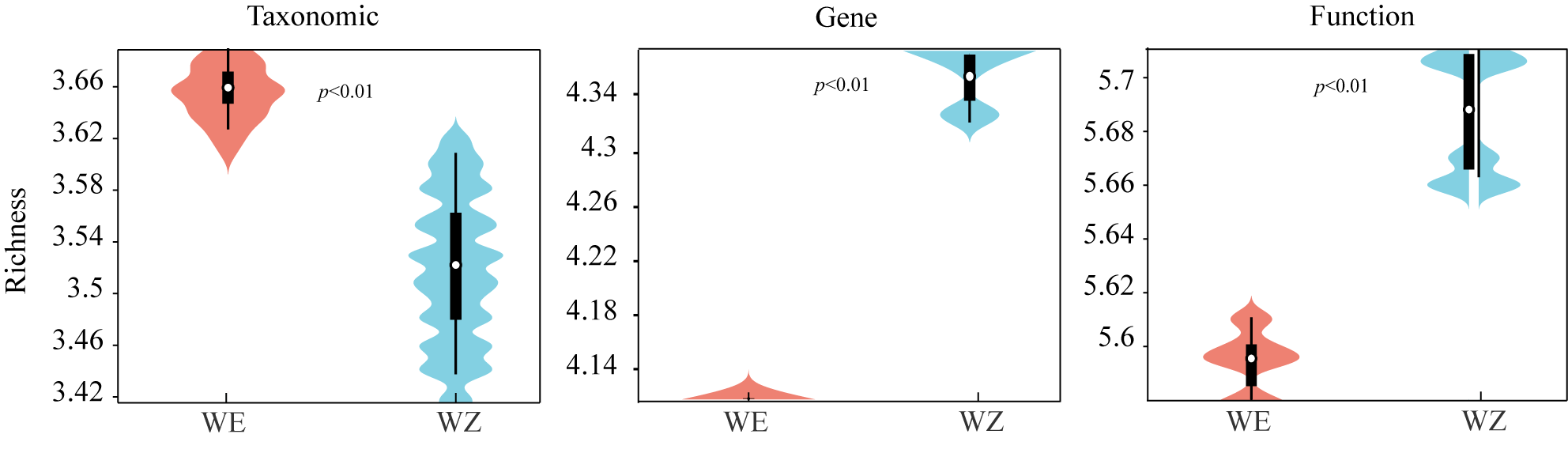


**Fig. S4** Comparison of richness between the water-land ecotone (WE) and the water-body zone (WZ).

**Fig. S5** Heatmap of Pearson's rank correlations coefficients among dissimilatory nitrate reduction to ammonium (DNRA), anammox and denitrifying genes before **(a)** and after **(b)** enrichment. The correlation coefficients are indicated by hue together with the corresponding *p* values (* *p* < 0.05; ** *p* < 0.01).


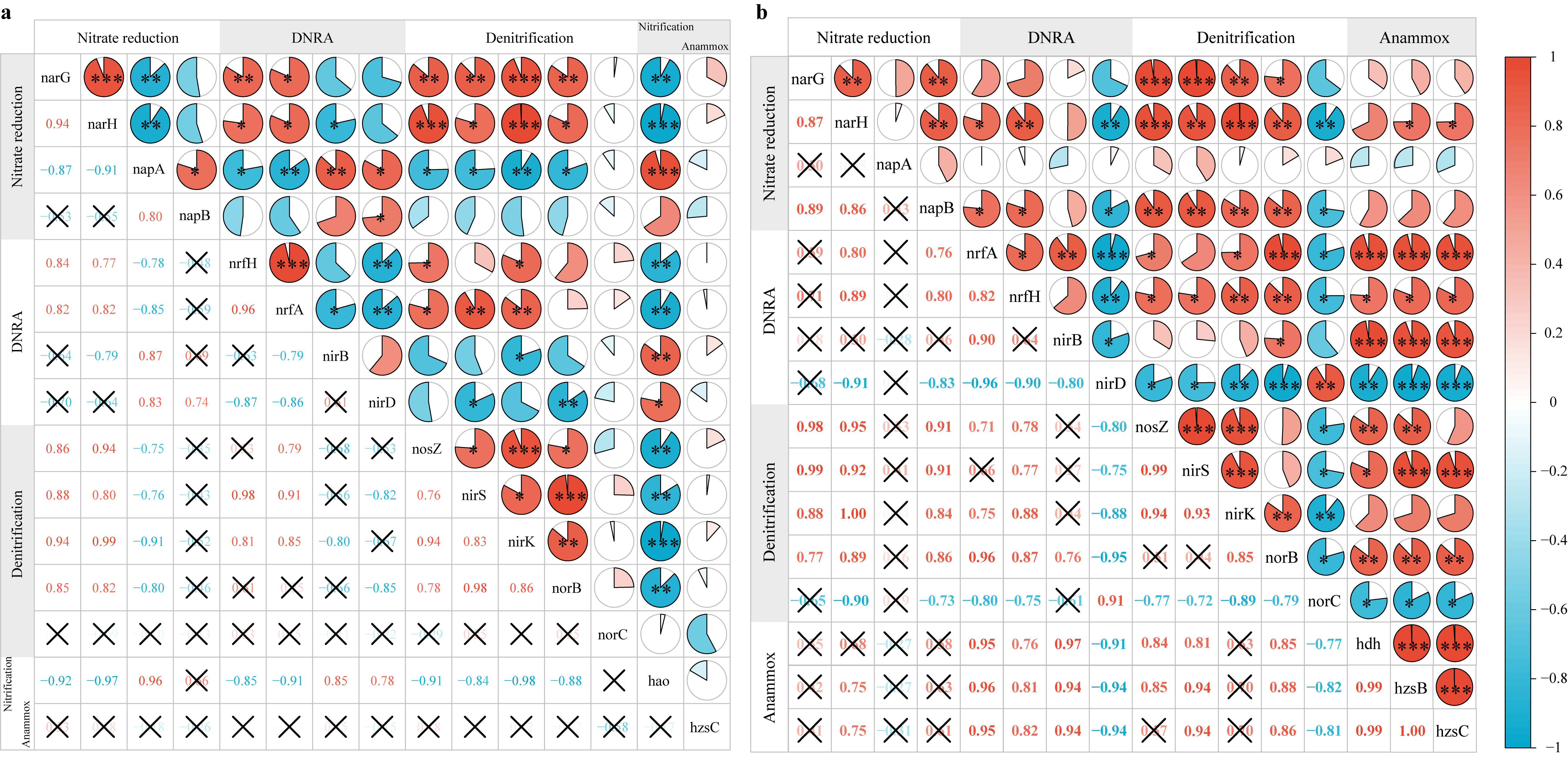


Water-land ecotone (WE), Water-body zone (WZ)


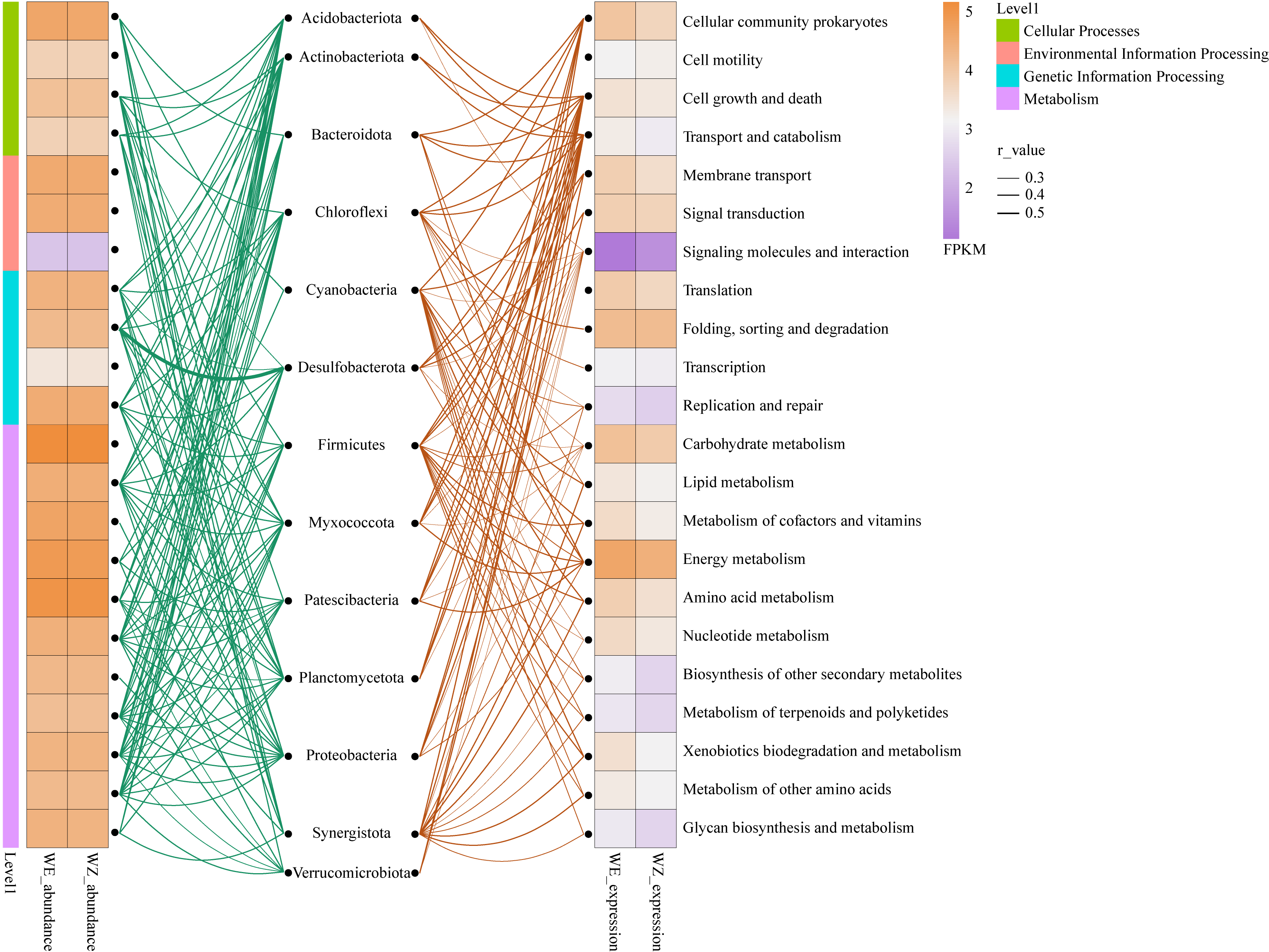


**Fig. S6** Metabolism pathways of KEGG modules showed by the gene abundance and gene expression in the two sites. For each functional pathway, the relative abundance was calculated as the sum of normalized marker pathway coverage. Pairwise Spearman’s correlation matrix summarized the relationships between bacteria and metabolism pathways determined by the Mantel tests.

**
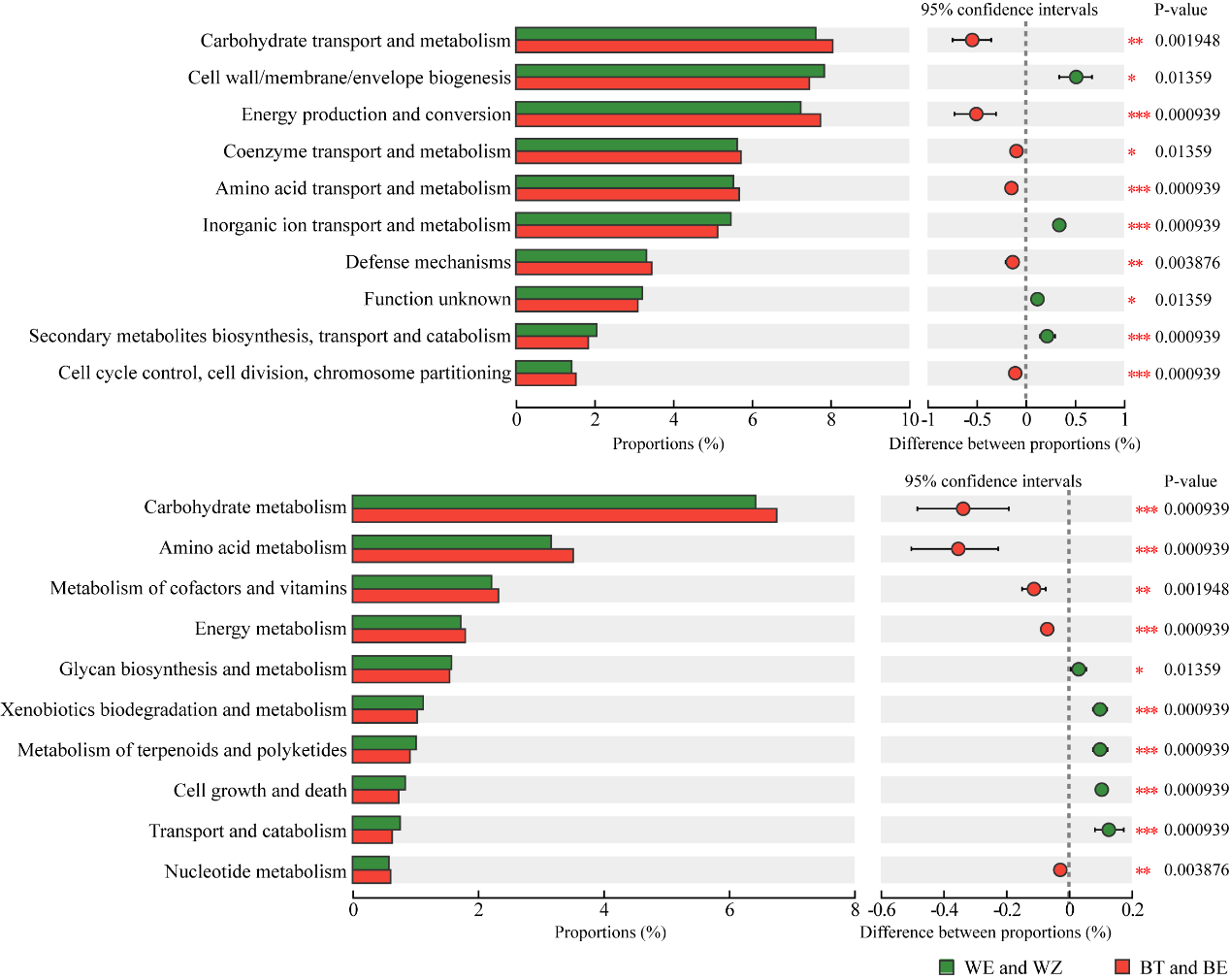
**

**Fig. S7** Comparing differences and abundance in KEGG and COG metabolic pathways before and after enrichment using Fisher’s exact test.


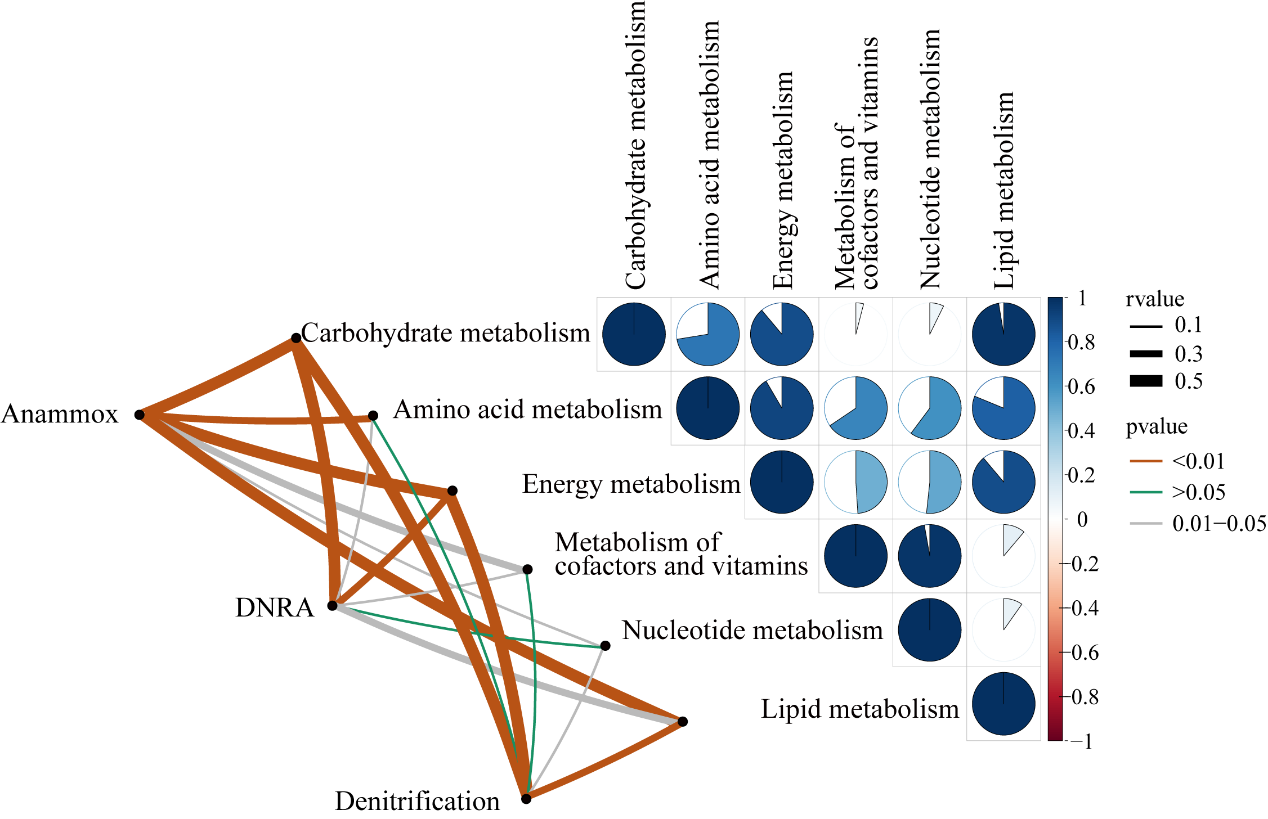


**Fig. S8** Metabolic pathways driving N cycling gene during enrichment bioreactor transition (BT) and bioreactor enrichments (BE). Pairwise Spearman’s correlation matrix of the metabolic pathways was shown with pie charts, and denitrification, dissimilatory nitrate reduction to ammonium (DNRA) and anammox of genes’ relationships to each metabolic pathways were determined by Mantel tests.


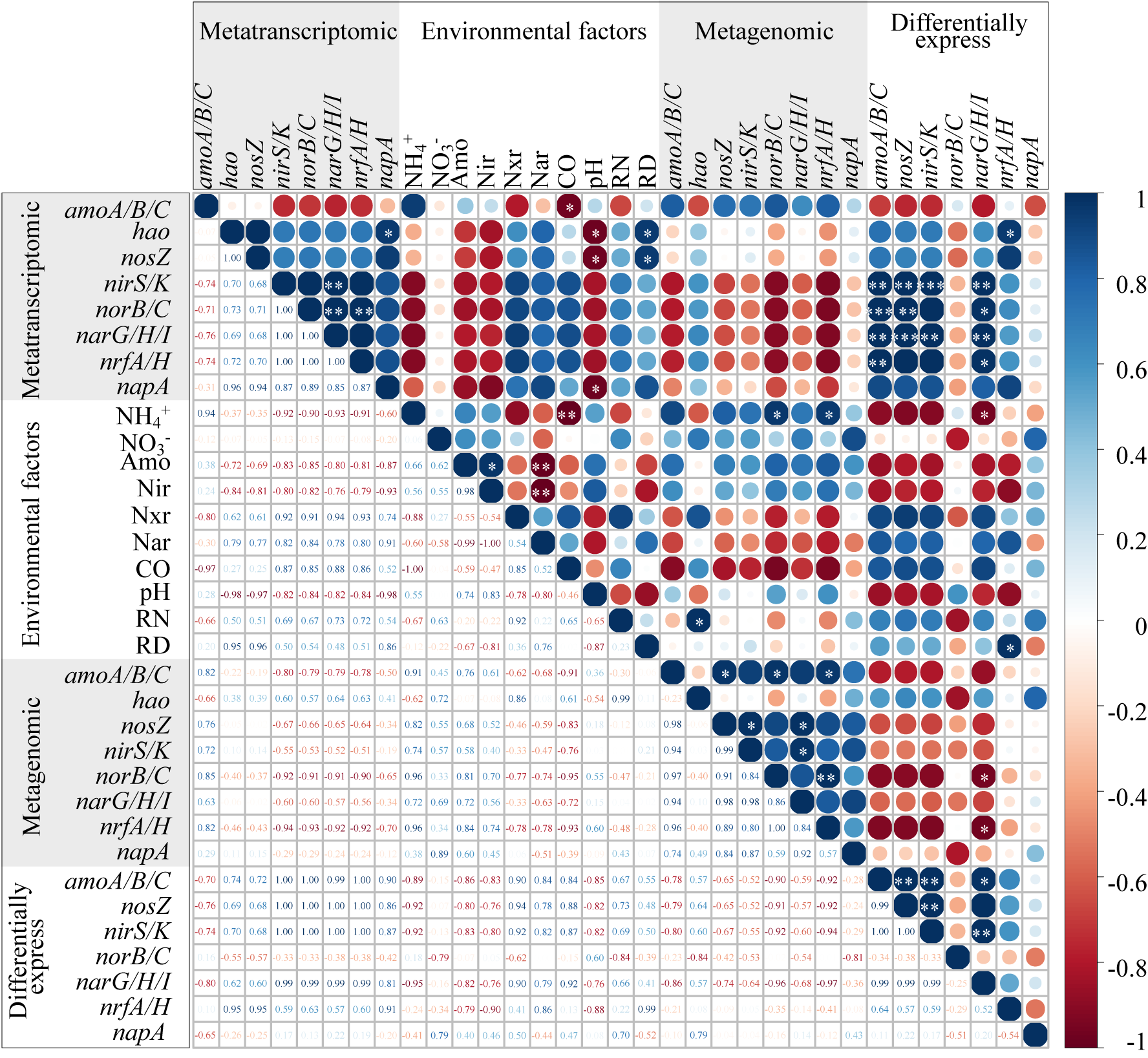


**Fig. S9** The correlation analysis based on the Pearson for the relationships between environmental factors and N cycling related genes abundance and expression. All the asterisks denote the significance of correlations (* *p* < 0.05, ** *p* < 0.01, and *** *p* < 0.001).


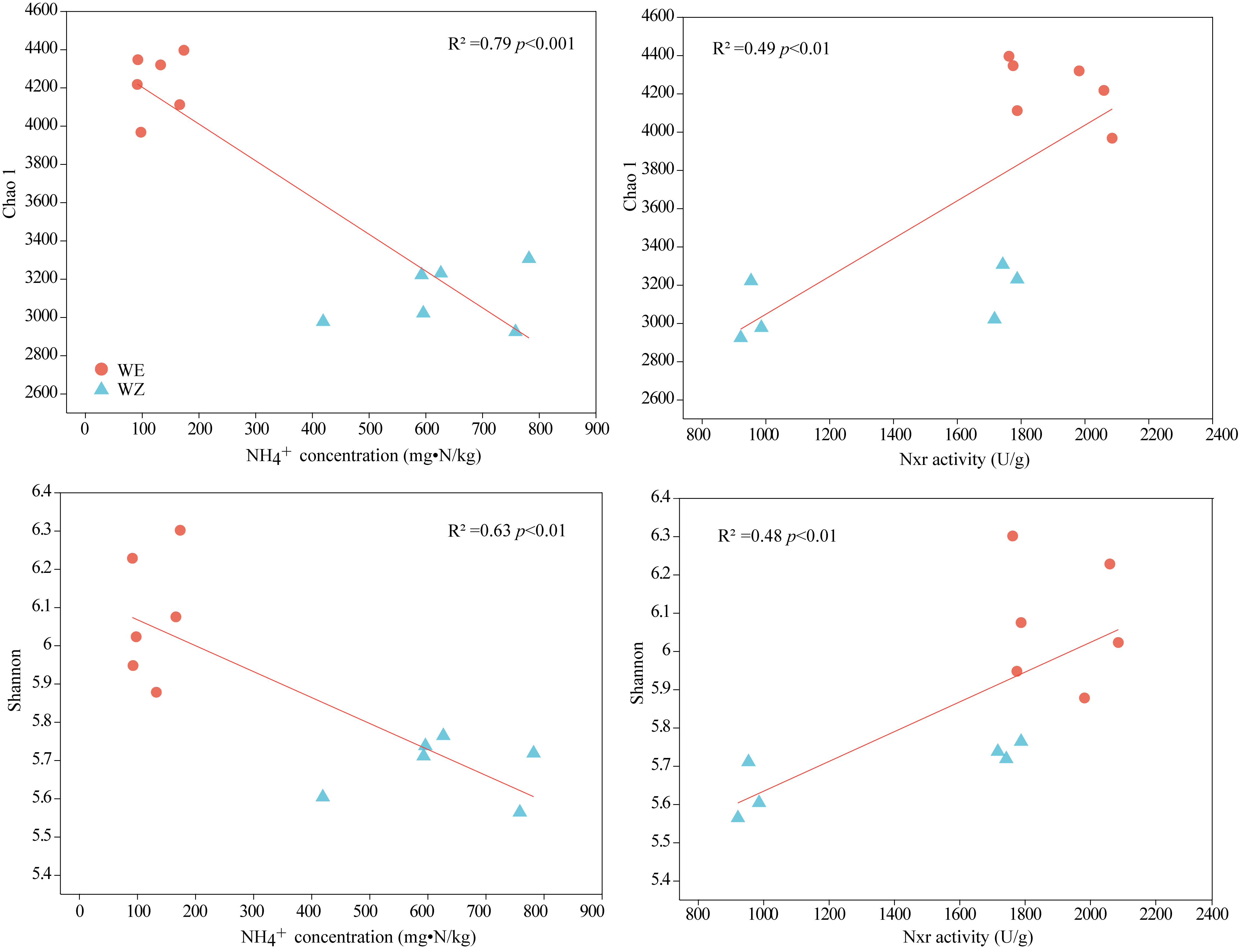


**Fig. S10** The linear regression analysis between environmental factors and alpha-diversity of bacterial community.

**
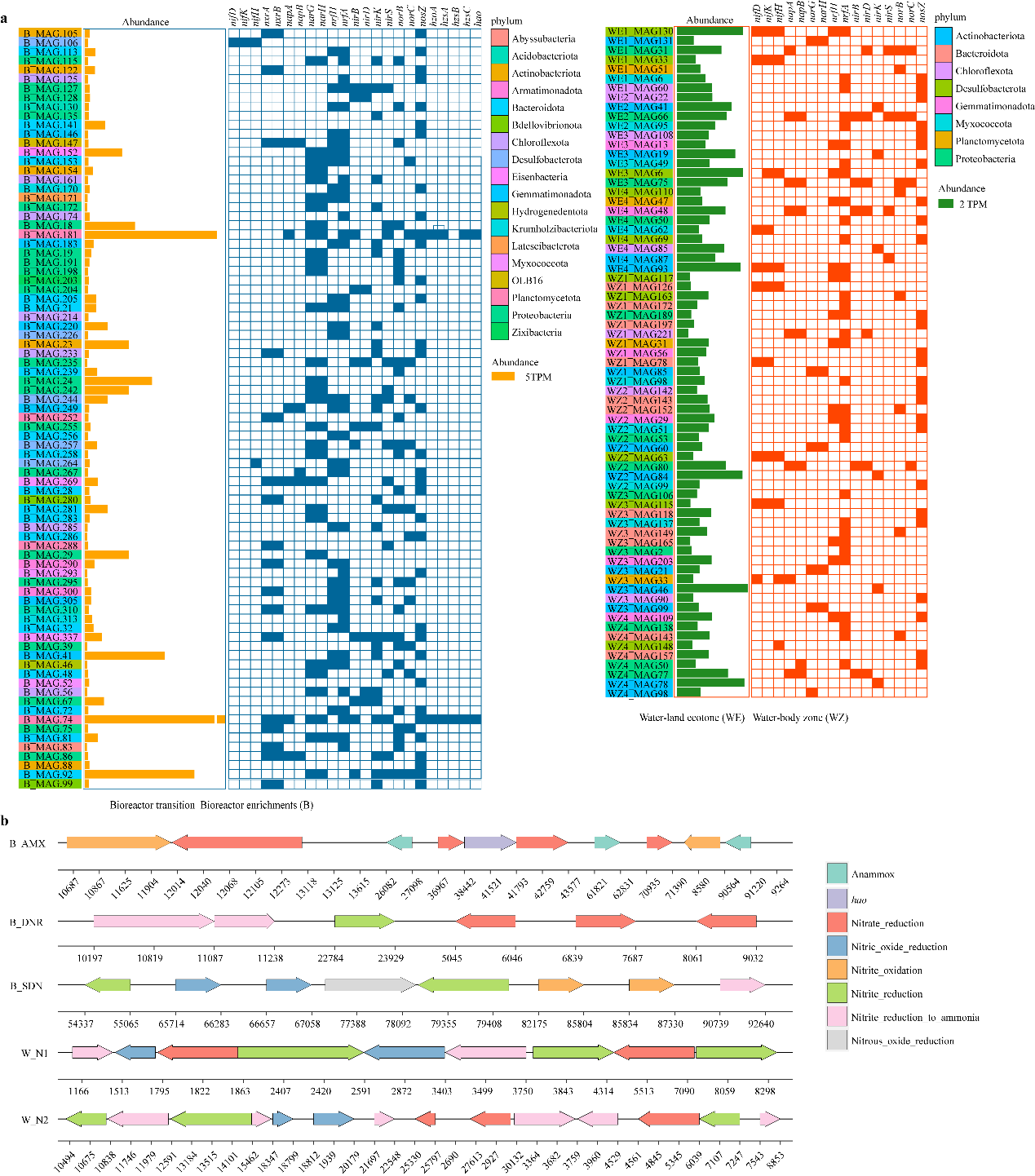
Fig. S11** The major metagenome-assembled genomes (MAGs) responsible for nitrogen (N) removal before and after enrichment. **(a)** Summary of high quality MAGs carrying N removal genes. The bar plot showed the relative abundance of each MAG before and after enrichment. The presence (colored) and absence (blank) of protein-encoding genes are given in the heatmap. The relative abundance of the recovered MAGs was calculated using CoverM. **(b)** Gene distribution of MAGs.


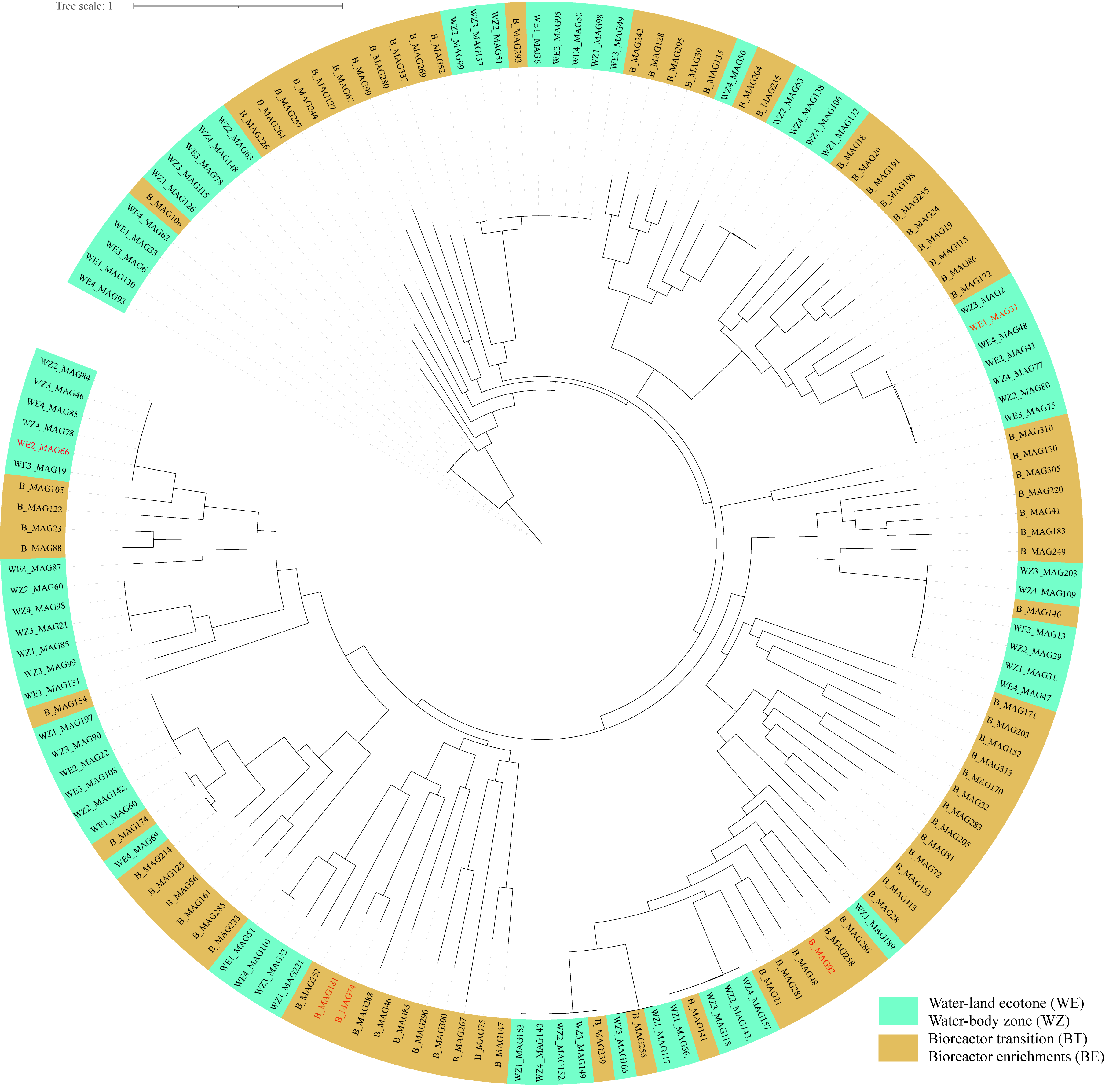


**Fig. S12** Phylogenetic distribution of metagenome-assembled genomes (MAGs) before and after the enrichment.


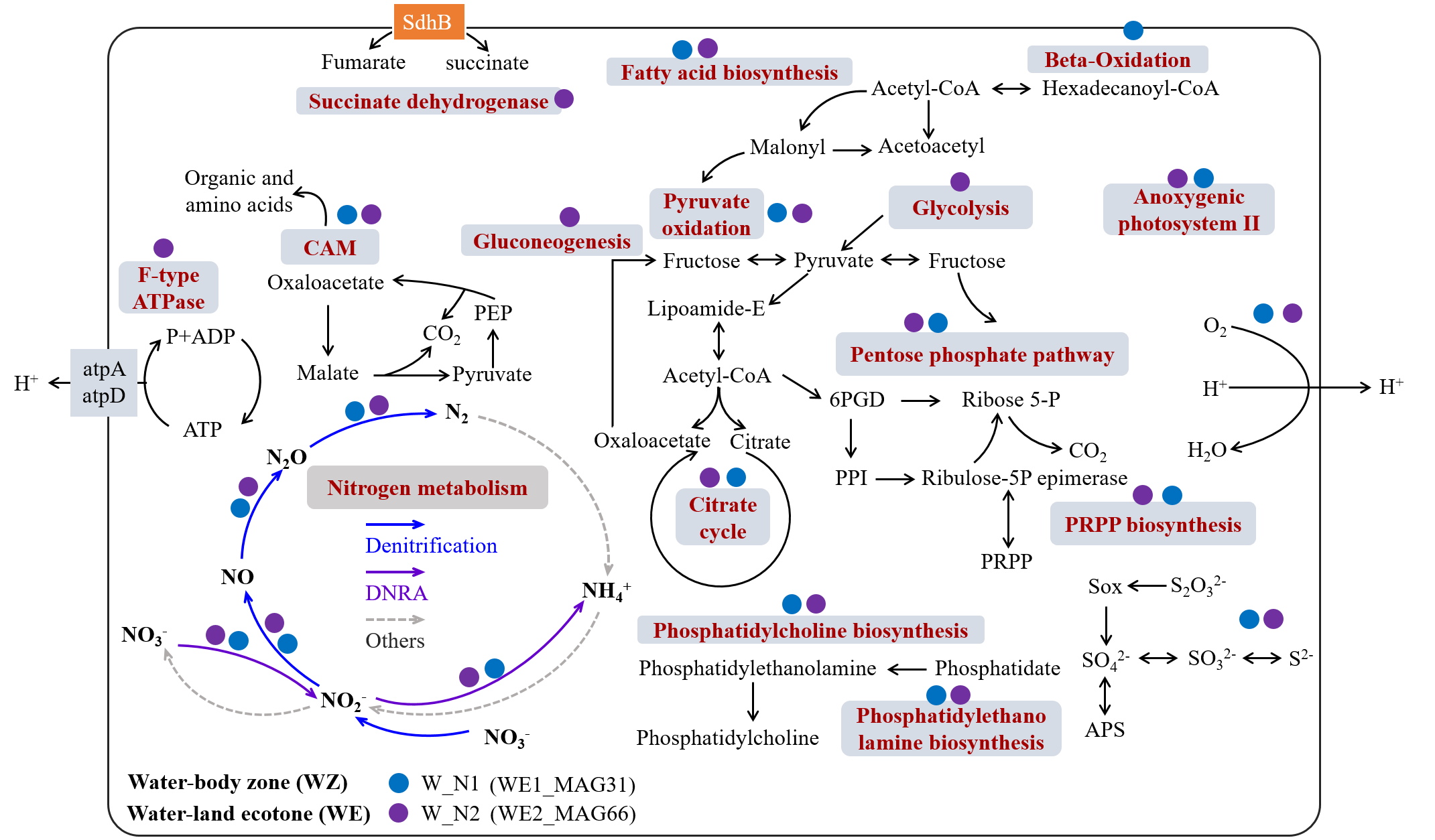


**Fig. S13** Potential metabolism pathways of nitrogen (N) removal coupled by the representative metagenome-assembled genomes (MAGs) in each process before enrichment.

Additional file 1

# 1.1 Experimental design of anammox enrichment in bioreactors with sediments

To gain a better understanding of microbial N removal function and adaptability, we manipulated the bioreactor by employing stable NH_4_^+^ and NO_2_^-^ to simplify environmental effects. The enrichment of anammox bacteria were used to provides further insights into understanding the microbial N removal functions. The anaerobic reactor (5 L) used for enrichment was set up by using the equally mixed sediments collected from WE and WZ. To improve the microbial attachment, polyurethane sponge filler was added, and water was circulated clockwise at 60 r/min. Anoxic condition in the reactor was maintained by continuously flushing with argon gas for 30 minutes before every experimental operation, and the reactor was covered with tin foil paper. To effectively enrich the targeted anammox bacteria, the influent of reactor was given a consistent NH_4_^+^ and NO_2_^-^ (60 mg N/L of each). The inorganic compounds also including CaCl_2_·2H_2_O (0.14 g/L), KH_2_PO_4_ (0.03 g/L), FeSO_4_·7H_2_O (9.00 mg/L) and MgCl_2_ (0.26 g/L). The NaHCO_3_ was added to control the pH (7.4-7.8), and the hydraulic retention time (HRT) for the reactor was kept at 48 h. The biotic and abiotic characteristics were monitored by analyzing the monthly collected samples during the 317 days enrichment.

**1.2 The rates of major N transformations analysis**

To evaluate the contributions of different pathways to the N removal, we measured the rates of major N transformations. The N reaction rates were measured using the ^15^NO_3_^-^ isotope pairing method as described previously (Yang et al., 2020). Briefly, fresh sediments were transferred into argon-flushed glass vials (20 mL), and pre-incubated at 33°C for 48 hours to remove residual NO_x_^-^ and oxygen. Then, a ^15^NO_3_^-^ stock solution (^15^N at 99.0%) was added to the pre-incubated solution to obtain a final concentration of 100 µmol/L of ^15^N. The pre-treated solution was then incubated at 33°C for 48 hours, and the incubation was stopped by adding a 7 mol/L ZnCl_2_ solution. The samples with isotope labelled N were transported on ice to the Third Institute of Oceanography, Ministry of Natural Resources for N_2_ measurement. An addition of NH_4_^+^ was used to confirm the presence of the anammox process. DNRA, denitrification, and anammox rates were measured in four technical replicates as the methods described previously (Xia et al., 2017).

**1.3 Sequencing analysis of the 16S rRNA gene**

The qPCR results showed a clear enrichment of anammox bacteria after 153 days, and then reached the highest level at 317 days. Thus, we selected the samples collected from the reactor at 153 and 317 days, as well as those before incubation (i.e., *in situ* sediments of WE and WZ, recorded as 0 day) for sequencing the 16S rRNA gene. The V3-V5 regions were amplified using 10 U of Phusion High-Fidelity DNA Polymerase (NEB, Inc, USA), 0.2 mM of each primer (Table S1), and 10 ng of template DNA. The amplified PCR products were determined by the TruSeq® DNA PCR-Free Sample Preparation Kit (Illumina, USA), then combined equally and mixed fully. The constructed libraries were sequenced on an Illumina HiSeq PE250 platform (Illumina, Inc., CA, USA) in Majorbio Bio-pharm Technology Co., Ltd (Shanghai, China). The amplicon sequencing data was analyzed as the method described previously (Zhang et al., 2022). Briefly, we used Trimmomatic v0.33 for quality control (Bolger et al., 2014), and removed sequences without primer fragments using FASTX_Toolkit, the sequences containing any ambiguous base ('N') were also removed. Chimeric sequences were identified and removed using UCHIME. FrameBot was used to correct frameshifts caused by sequence errors when generating OTUs using Uparse cluster.

**References**

Yang, Y.C., Pan, J., Zhou, Z.C., Wu, J.P., Liu, Y., Lin, J.G., Hong, Y.G., Li, X.Y., Li, M. and Gu, J.D. (2020). Complex microbial nitrogen-cycling networks in three distinct anammox-inoculated wastewater treatment systems. *Water Research*, *168*, 115142. <https://doi.org/10.1016/j.watres.2019.115142>.

Xia, X.H., Jia, Z.M., Liu, T., Zhang, S.B. and Zhang, L.W. (2017). Coupled Nitrification-Denitrification Caused by Suspended Sediment (SPS) in Rivers: Importance of SPS Size and Composition. *Environmental Science & Technology*, *51*, 212-221. <https://doi.org/10.1021/acs.est.6b03886>.

Zhang, D.D., Li, M.Y., Yang, Y.C., Yu, H., Xiao, F.S., Mao, C.Z., Huang, J., Yu, Y.H., Wang, Y.F., Wu, B., Wang, C., Shu, L.F., He, Z.L. and Yan, Q.Y. (2022). Nitrite and nitrate reduction drive sediment microbial nitrogen cycling in a eutrophic lake. *Water Research*, *220*, 118637. <https://doi.org/10.1016/j.watres.2022.118637>.Bolger, A.M., Lohse, M. and Usadel, B. (2014). Trimmomatic: a flexible trimmer for Illumina sequence data. *Bioinformatics*, *30*, 2114-2120. <https://doi.org/10.1093/bioinformatics/btu170>.
